# Highly Efficient Manganese Bromides with Reversible Luminescence Switching through Amorphous–Crystalline Transition

**DOI:** 10.1021/acsami.4c09396

**Published:** 2024-10-03

**Authors:** Guang-Hsun Tan, Hao-Cheng Lin, Hao-Chi Liang, Chih-Wen Pao, Po-Yu Chen, Wei-Tsung Chuang, Chung-An Hsieh, Dalia M. Dorrah, Ming-Chia Li, Li-Yin Chen, Ho-Hsiu Chou, Hao-Wu Lin

**Affiliations:** † Department of Materials Science and Engineering, National Tsing Hua University, Hsinchu 30013, Taiwan; ‡ Department of Chemical Engineering, National Tsing Hua University, Hsinchu 30013, Taiwan; § National Synchrotron Radiation Research Center, Hsinchu 30076, Taiwan; ∥ Advanced Packaging Instrumentation and Metrology Laboratory, Industrial Technology Research Institute, Hsinchu 30013, Taiwan; ⊥ Department of Photonics, College of Electrical and Computer Engineering, National Yang Ming Chiao Tung University, Hsinchu 30010, Taiwan; # Department of Biological Science and Technology, College of Biological Science and Technology, National Yang Ming Chiao Tung University, Hsinchu 30010, Taiwan; ∇ Center for Intelligent Drug Systems and Smart Bio-devices (IDS2B), Hsinchu 30068, Taiwan

**Keywords:** manganese(II) bromides, stimuli-responsive materials, reversible luminescence switching, time−temperature indicator, X-ray scintillator

## Abstract

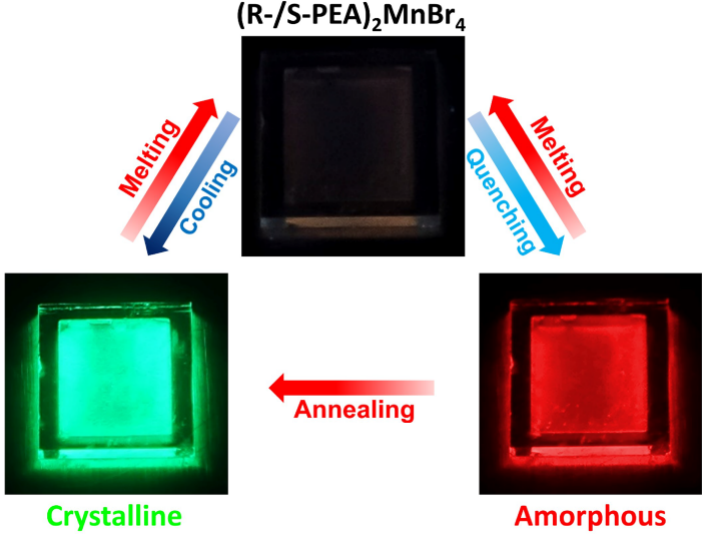

While luminescent stimuli-responsive materials (LSRMs) have become one of the most sought-after materials owing to their potential in optoelectronic applications, the use of earth-scarce lanthanides remains a crucial problem to be solved for further development. In this work, two manganese-based LSRMs, (*R*)-(+)-1-phenylethylammonium manganese bromide, (R-PEA)_2_MnBr_4_, and (*S*)-(−)-1-phenylethylammonium manganese bromide, (S-PEA)_2_MnBr_4_, are successfully demonstrated. Both (R-PEA)_2_MnBr_4_ and (S-PEA)_2_MnBr_4_ show a kinetically stable red-emissive amorphous state and a thermodynamically stable green-emissive crystalline state at room temperature, where the fully reversible transition can be done through melt-quenching and annealing processes. Based on this property, a reusable manganese-halide-based time–temperature indicator is demonstrated for the first time. Furthermore, an X-ray scintillator with a low limit of detection (18.1 nGy/s) and a high spatial resolution limit (30.0 lp/mm) are achieved by exploiting the high transparency of amorphous states. These results uncover the multifunctionality of manganese halides and pave the way for upcoming research.

## 1 Introduction

Stimuli-responsive materials (SRMs) refer to a class of materials that can convert external stimuli like heat, light, pressure, or solvent into changes in dielectric constant, absorption, luminescence, and other physical or chemical properties.^1^ Among all SRMs, luminescent stimuli-responsive materials (LSRMs) have drawn great attention and have become the building blocks of sensors,^2^ logic gates,^3^ and anticounterfeiting technology^4^ nowadays. Nevertheless, most of the LSRMs rely on lanthanides, which are earth-scarce and expensive.^5^ Therefore, it is necessary to find earth-abundant and inexpensive alternatives for the further development of LSRMs.

Recently, organometallic halide perovskites (OMHPs) have garnered global attention owing to their low cost and excellent performance in optoelectronic devices.^6^ The soft lattice and adjustability of OMHPs from 3D structures to lower dimensional 2D, 1D, or even 0D structures via manipulation of composition stoichiometry or external stimuli make them suited for serving as next-generation LSRMs.^7^ However, the toxicity of Pb^2+^ in traditional OMHPs has prompted researchers to seek eco-friendly alternatives, such as Sn^2+^, In^3+^, Bi^3+^, Cu^+^, Zn^2+^, and Mn^2+^, to name just a few.^8−12^ Especially, Mn^2+^-based metal halides have been widely studied over the past five years due to their earth-abundant and nontoxic nature, mutable luminescence wavelength, excellent efficiency, outstanding thermal-quenching resistance, facile synthesis processes, and diverse light-emitting applications including light-emitting diodes and scintillators.^13−17^ Additionally, the emission from the d–d transition ^4^T_1_ (G) to ^6^A_1_ (S) of Mn^2+^ strongly depends on the ligand field and is sensitive to the coordination environment, making it stimuli-responsive and applicable for sensors.^18−21^ For instance, [MnBr_2_(dppeO_2_)] prepared by Wu et al. can switch from green to red emissive states upon exposure to *N*,*N*-dimethylformamide vapor.^18^ Liu et al. reported green emissive *trans*-2,5-dimethylpiperazine manganese(II) bromide (C_6_N_2_H_16_)MnBr_4_, which can transform to a nonemissive state by absorbing acetone molecules.^19^ Similar results were reported by Knog et al. and Xue et al. as well.^20,21^

Apart from the above-mentioned solvatochromic process, thermochromism also plays an important role in SRMs, as temperature is a more general and accessible physical parameter in our daily lives. Peng et al. synthesized C_4_H_14_N_2_MnBr_4_ with orange–green dual emission bands between 80 and 490 K,^22^ Zhang et al. proposed [TMPA]_2_MnI_4_ that undergoes an orange–green transition from 308 to 383 K,^23^ and Liu et al. even reported thermally robust Rb_2_MnBr_4_(H_2_O)_2_ with reversible luminescence transition from red RbMnBr_3_ at 403 K to green Rb_3_MnBr_5_ at 473 K.^24^ Despite these promising results, all of the published thermochromic manganese halides are volatile temperature sensors that return to their initial emission wavelength once cooled down, which is unable to record the thermal history and act as indicators for time-and-temperature-sensitive products.

In this work, inspired by the design strategy of state-of-the-art functional manganese halide materials,^25,26^ manganese-based LSRMs showing notable absolute photoluminescence quantum yields (PLQYs) were successfully synthesized by reacting (*R*)-(+)-1-phenylethylammonium bromide (R-PEABr) or (*S*)-(−)-1-phenylethylammonium bromide (S-PEABr) with manganese bromide through a solvent-free mechanochemical process. (R-PEA)_2_MnBr_4_ or (S-PEA)_2_MnBr_4_ (abbreviated as (R-/S-PEA)_2_MnBr_4_) exhibits two distinct emission states at room temperature, which are a kinetically stable red-emissive amorphous state (λ_max_ = 648 nm) and a thermodynamically stable green-emissive crystalline state (λ_max_ = 526 nm). The transition between these two states is fully reversible via the melt-quenching and annealing processes, and the ratio of transition can be controlled by the annealing time. Making use of this advantage, an unprecedented reusable manganese-halide-based time–temperature indicator (TTI) was demonstrated. Considering the low toxicity, earth abundance, and the facile melt-quenching fabrication process compared to the traditional CsI:Tl scintillator, an X-ray scintillator with an outstanding spatial resolution limit of 30.0 lp/mm and a low limit of detection (LoD) of 18.1 nGy/s was achieved as well by exploiting the high transparency of amorphous states. The intriguing properties of (R-/S-PEA)_2_MnBr_4_ uncover the multifunctionality of manganese halides and set the stage for upcoming research.

## 2 Experimental Methods

### 2.1 Materials

Manganese(II) bromide (MnBr_2_, 99%) was purchased from Acros. (*S*)-(−)-1-Phenylethylamine (98%) and (*R*)-(+)-1-phenylethylamine (98%) were purchased from Thermo Fisher. All of the materials were used as received without purification. S-PEABr and R-PEABr were synthesized according to previous literature,^27^ and the purity of the final products was confirmed with elemental analyses (S-PEABr calcd (%) C 47.55, H 5.99, N 6.93; found (%) C 47.79, H 5.86, N 6.87. R-PEABr calcd (%) C 47.55, H 5.99, N 6.93; found (%) C 47.61, H 5.84, N 6.83).

### 2.2 Synthesis of (R-/S-PEA)_2_MnBr_4_ Powder

R-/S-PEABr (808.36 mg, 4 mmol) and MnBr_2_ (429.49 mg, 2 mmol) were mixed with ZrO_2_ milling balls (diameter: 3 and 5 mm) at a ball-to-reagent ratio of 4 inside a glass milling jar. The jar was then mounted on a planetary ball mill machine and rotated at 800 rpm for 3 h. The whole process was completed in a N_2_-filled glovebox.

### 2.3 Synthesis of (R-PEA)_2_MnBr_4_ Single Crystals

Ball-milled (R-PEA)_2_MnBr_4_ powders were first melted completely at 160 °C, followed by gradual cooling to room temperature on the hot plate overnight. The whole process was completed in a N_2_-filled glovebox.

### 2.4 Synthesis of PDMS/(S-PEA)_2_MnBr_4_ Films

Ball-milled (S-PEA)_2_MnBr_4_ powders were mixed with PDMS precursors through hand stirring, followed by polymerization at 60 °C for 1 h. The whole process was completed in a N_2_-filled glovebox.

### 2.5 Fabrication of Scintillator Sample

First, ball-milled (R-/S-PEA)_2_MnBr_4_ powders and spacers (thickness: 170 μm) were placed between two glass substrates and melted completely at 160 °C. Subsequently, the sample was degassed under vacuum for 1 h and quenched to room temperature. The whole process was completed in a N_2_-filled glovebox.

### 2.6 General Characterization

Thermogravimetric analysis (TGA) and differential scanning calorimetry (DSC) above room temperature were measured using a METTLER TOLEDO TGA/DSC 2 at a heating rate of 10 °C/min under constant N_2_ flow. Low-temperature differential scanning calorimetry (LTDSC) was acquired using a Netzsch DSC 204 F1 with N_2_ purging. Single-crystal X-ray diffraction (SCXRD) was analyzed using an XtaLAB Synergy DW diffractometer with a Cu Kα (λ = 1.54 Å) source. The crystal temperature was controlled at 100 K throughout the data collection process, and the crystal structure was visualized with Vesta.^28^ Powder X-ray diffraction (PXRD) measurements were completed at beamline 01C2 of the Taiwan Light Source with 12 keV radiation. Absorption spectra were measured with an ultraviolet–visible spectrophotometer (Shimadzu UV2600). Photoluminescence excitation (PLE) spectra were acquired using a HORIBA Fluoromax Plus. Photoluminescence (PL) spectra and photoluminescence quantum yields (PLQYs) were measured using a homemade system comprising a calibrated integrating sphere (Labsphere), a spectrometer (Ocean Optics FLAME-S-UV–VIS), and a stable 365 nm excitation source in an N_2_-filled glovebox. Time-resolved photoluminescence (TRPL) measurements were performed using Becker and Hickl PMS-400A and MSA-300. Circularly polarized luminescence (CPL) and circular dichroism (CD) measurements were conducted using JASCO CPL-300 and J-1700, respectively. Extended X-ray absorption fine structure (EXAFS) measurements were completed at the beamline 44A of the Taiwan Photon Source, and the data were processed with ATHENA and fitted with ARTEMIS.^29^

### 2.7 X-ray Scintillator Measurements

The attenuation efficiency was obtained using the following equation:^30^
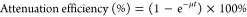
1

where μ is the product of the attenuation coefficient from the XCOM database and the density of the material, and *t* refers to the thickness of the sample. The limit of detection (LoD) was measured with a photomultiplier tube (Becker and Hickl PMC-100-1) combined with a single photon counter (Stanford Research Systems SR400). The scintillator images were taken with a camera (Sony ZV-E10). The line pair patterns were obtained using the standard resolution line pair card (Fluke Biomedical, 07-525).

## 3 Result and Discussion

### 3.1 Structural and Thermal Properties

**Figure 1:**
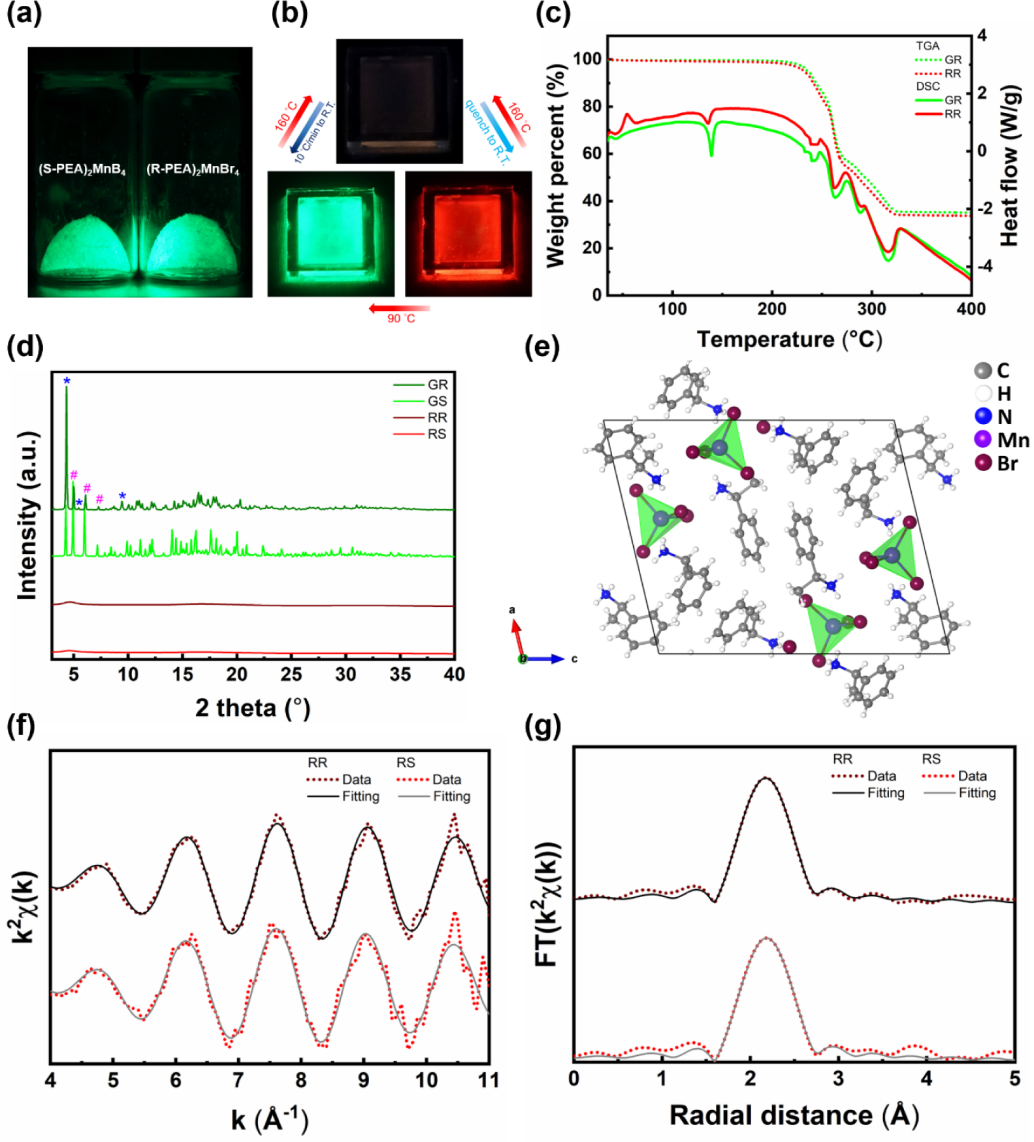
(a) The appearance of ball-milled (R-/S-PEA)_2_MnBr_4_ powder under UV light illumination. (b) The transition process between different states of (R-/S-PEA)_2_MnBr_4_. (c) TGA–DSC measurement of GR and RR. (d) XRD of GR, GS, RR, and RS. Hash signs and asterisk signs specify the diffraction peaks from orthorhombic structure and monoclinic structure, respectively. (e) The crystal structure of monoclinic GR. (f) The *k*^2^χ(*k*) spectra and (g) the Fourier-transformed *k*^2^χ(*k*) spectra of RR and RS.

The synthesis process of (R-/S-PEA)_2_MnBr_4_ powder is depicted in Figure S1. Briefly, R-/S-PEABr (Figure S1a) and MnBr_2_ (molar ratio 2:1) were carefully weighed and loaded into a milling jar containing zirconium oxide (ZrO_2_) milling balls (Figure S1b). The jar was rotated at 500 rpm for 4 h on a planetary ball mill machine, and powders showing green PL under UV light excitation were successfully synthesized (Figure 1a). (R-/S-PEA)_2_MnBr_4_ exhibits a low melting point property. At 160 °C, (R-/S-PEA)_2_MnBr_4_ can be readily melted, resulting in a transition of the PL to a faint orange. Surprisingly, upon quenching the (R-/S-PEA)_2_MnBr_4_ liquid to room temperature, the PL changes strikingly and turns into a kinetically stable state with vivid red emission. Alternatively, if the melt is slowly cooled to room temperature (10 °C/min) or annealed at 90 °C after quenching, the PL converts to its thermodynamically stable bright green state (Figure 1b). It should be noted that the whole process is fully reversible. Moreover, the appearances of the green-emissive (R-/S-PEA)_2_MnBr_4_ (abbreviated as GR/GS) and red-emissive (R-/S-PEA)_2_MnBr_4_ (abbreviated as RR/RS) states are quite distinct. The GR/GS state is characterized by large and directional grains, whereas the RR/RS state presents a transparent and glassy texture (Figure S2).

In order to gain deeper insight into the transition between GR–RR and GS–RS states, DSC and TGA were first conducted. As displayed in Figure 1c, an exothermic peak around 55 °C for RR corresponds to the crystallization process. The endothermic peak near 139 °C for both the GR and RR reveals the melting point of these two states. Similar results were obtained for GS and RS as well (Figure S3). Additionally, the melting point of R-PEABr and S-PEABr was found to be 122 °C, indicating that the low melting point of (R-/S-PEA)_2_MnBr_4_ may be related to the property of the precursor (Figure S4). PXRD and SCXRD were completed to study the detailed crystal structures, and the apparent difference in crystallinity between GR/GS and RR/RS was discovered (Figures 1d and S5). Clear diffraction peaks of GR and GS were observed, which can be linked to their high crystallinity. It is noteworthy that while peaks at 5.01°, 6.08° and 7.29° (hash sign) can be assigned to the orthorhombic *P*2_1_2_1_2_1_ space group reported by Cai et al. (CCDC 2122231),^31^ those at 4.33°, 5.46°, and 9.43° (asterisk sign) originate from the monoclinic *P*2_1_ space group (check Table S1 for detailed crystallographic data, Figure S6 for calculated diffraction patterns, and Figure S7 for zoomed-in diffraction patterns), which has the same tetrahedral [MnBr_4_]^2–^ structure as its orthorhombic counterpart but a slightly distorted lattice (Figure 1e). The detailed Mn–Br bond lengths and bond angles are listed in Table S2. Furthermore, as can be observed in the temperature-dependent XRD (Figure S8), the diffraction peak at 4.33° disappears after the temperature increases, indicating that the monoclinic *P*2_1_ structure is metastable and will transform to the orthorhombic *P*2_1_2_1_2_1_ structure at elevated temperature. On the contrary, the lack of sharp and intense diffraction peaks for RR and RS implies their amorphous nature identical to the molten state. This point of view was further confirmed by LTDSC of RR and RS, where the glass transition temperature was determined to be 20 °C, lower than room temperature (Figure S9). Despite the fact that PL switching between crystalline and amorphous states has been reported in organic molecules^32^ and antimony halides,^33^ to our knowledge, this is the first example in manganese halides.

### 3.2 X-ray Absorption Spectroscopy

Given that the interatomic relationship of RR and RS cannot be solved directly by PXRD, Mn K-edge EXAFS was studied to attain more concrete evidence. In order to get the accurate amplitude reduction factor (), MnBr_2_ with known coordination numbers (CN) was chosen as the standard sample. The *k*^2^-weighted χ(*k*) in *k*-space ranging from 4 to 10 Å^–1^ was selected for the Fourier transform to R-space, and the Mn–Br along with Mn–Mn scattering was picked out by restricting the value of the radial distance between 1.85 and 5 Å for analysis. The experimental data along with fitting curves are displayed in Figure S10. Fitting parameters including the , the CN, the bond length (*D*), the Debye–Waller factor (σ^2^), and the *R*-factor obtained are listed in Table S3. RR and RS samples were measured and fitted afterward. Similarly, the *k*^2^-weighted χ(*k*) in *k*-space ranging from 4 to 10 Å^–1^ was selected for the Fourier transform to R-space, where the first shell of Mn–Br scattering was picked out by restricting the value of radial distance between 1.2 and 3.2 Å. The whole fitting process was conducted at fixed , and the experimental data along with fitting curves are displayed in Figure 1f,g. Fitting parameters are summarized in Table 1. The CNs of RR and RS are 5.62 and 6.14, respectively, which implies an octahedrally coordinated structure. In addition, *R*-factors lower than 0.02 are indicative of reliable fitting results.^34^ Accordingly, the transition between green and red emissive states involves the change of coordination number, similar to other manganese halides reported before.^24^

**Table 1:** Fitting Parameters Used in the EXAFS Analysis of RR and RS

Material		*N*	*r* (Å)	Δ*E*_0_ (eV)	σ^2^	*R*-factor
RR	0.59 (fixed)	5.62	2.51	0.87	0.0045	0.0015
RS		6.14	2.51	0.95	0.0053	0.0024

### 3.3 Photophysical Properties

**Figure 2:**
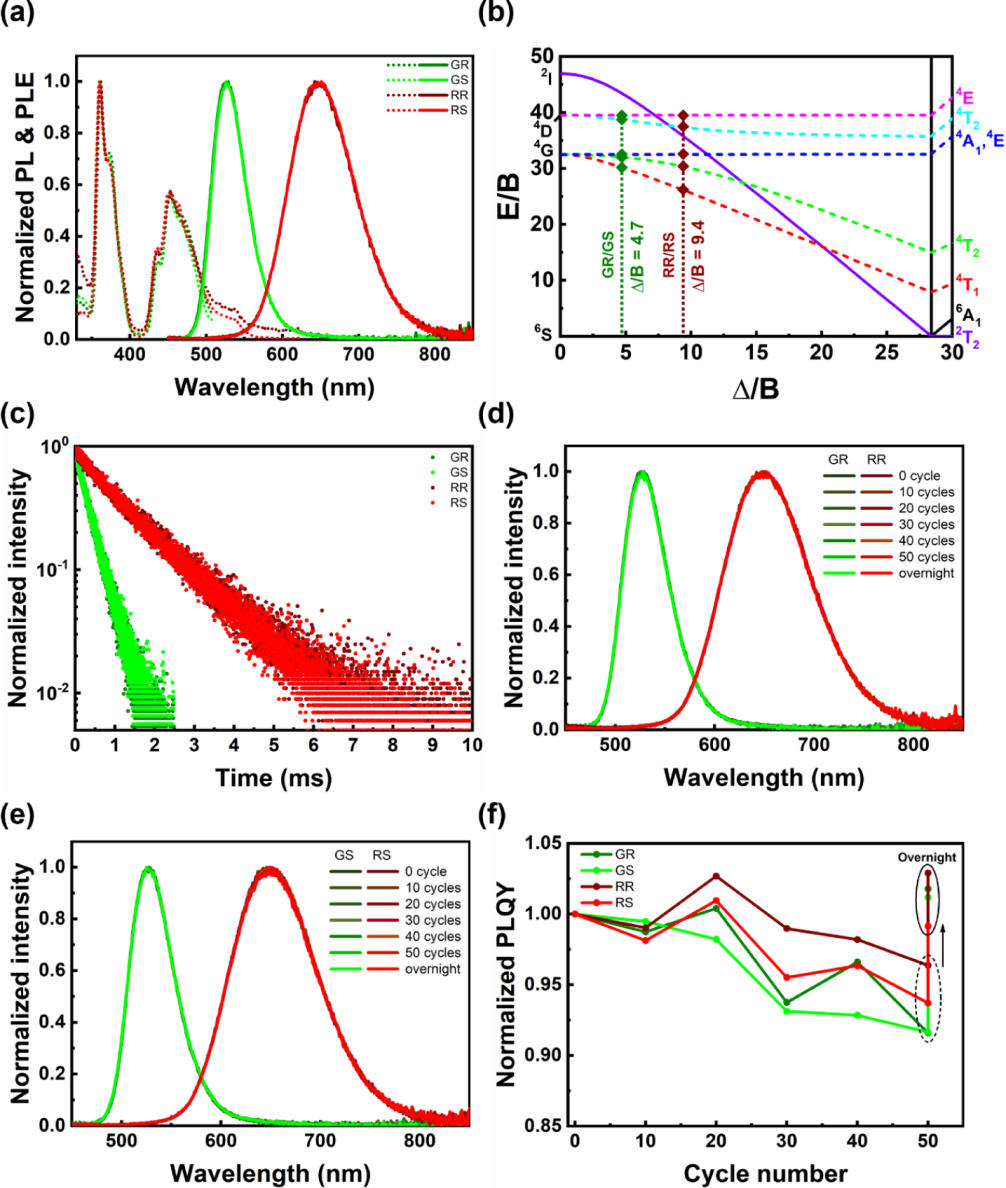
(a) The PLE (dashed line) and the PL (solid line) spectra of GR, GS, RR, and RS. (b) The d^5^ Tanabe–Sugano diagram. (c) The TRPL decay curves of GR, GS, RR, and RS. (d) The PL spectra of GR and RR and (e) GS and RS during thermal cycle measurements. (f) The normalized PLQY variation of GR, GS, RR, and RS during thermal cycle measurements.

The PL and PLE spectra of (R-/S-PEA)_2_MnBr_4_ are illustrated in Figure 2a. For GR and GS, the green emission with a peak wavelength at 526 nm and a full width at half-maximum (fwhm) of 52 nm is observed, which is in accordance with other tetrahedral manganese(II) bromides.^19^ For RR and RS, a typical red emission of octahedral manganese(II) bromides with a peak wavelength at 648 nm and an fwhm of 101 nm is dominant.^35^ The optimal absolute PLQYs of GR, GS, RR, and RS were determined to be 91.3, 98.6, 64.9, and 79.9%, respectively (Figure S11). To acquire detailed information such as ligand field splitting energy (Δ) and Racah parameter (*B*), PLE spectra were analyzed based on the d^5^ Tanabe–Sugano diagram. The peak at 470 nm (21 277 cm^–1^) of GR and GS along with that at 539 nm (18 553 cm^–1^) of RR and RS can be assigned to the ^6^A_1_ (S) → ^4^T_1_ (G) transition, while the remaining peaks at 453 nm (22 075 cm^–1^), 435 nm (22 989 cm^–1^), 372 nm (26 882 cm^–1^), and 360 nm (27 778 cm^–1^) can be attributed to ^6^A_1_ (S) → ^4^T_2_ (G), ^6^A_1_ (S) → ^4^A_1_, ^4^E (G), ^6^A_1_ (S) → ^4^T_2_ (D), and ^6^A_1_ (S) → ^4^E (D) transitions, respectively. Furthermore, the drastic increase below 300 nm is consistent with the absorption spectra of R-/S-PEABr, revealing the energy transfer process between organic cations and Mn^2+^ luminescent centers (Figure S12).^36^ By calculating the ratio of the energy between ^6^A_1_ (S) → ^4^T_1_ (G) and ^6^A_1_ (S) → ^4^T_2_ (G) transitions, Δ_GR_/*B*_GR_ = Δ_GS_/*B*_GR_ = 4.7 and Δ_RR_/*B*_RR_ = Δ_RS_/*B*_RS_ = 9.4 can be determined (Figure 2b). After reference to the Tanabe–Sugano diagram, values of *B* and Δ are estimated and listed in Table S4. The great discrepancy in Δ indicates that the coordination environment is substantially different, where the larger Δ of RR and RS can be associated with a higher coordination number since Br^–^ is the only Lewis base in this study.^37^ The discussion above is in line with the results of the TRPL analysis in Figure 2c. According to the Laporte rule and previous studies, Mn^2+^ in an octahedral field will possess a longer average lifetime (τ_a_) than in a tetrahedral field due to its centrosymmetric coordination structure.^38^ Therefore, the significantly longer τ_a_ of RR and RS (∼1.316 ms) over GR and GS (∼332.7 μs) emphasizes their octahedrally coordinated Mn^2+^ structure.

Considering the chirality of precursors, the CD and the CPL of GR, GS, RR, and RS were analyzed. It is presented clearly in Figure S13a that the CD spectra of RR and RS thick films are symmetrical, and peaks near 285 nm are indicative of the existence of R-PEA^+^ and S-PEA^+^. Similar results were observed in GR and GS powders, while the less symmetrical spectra may be due to the lower homogeneity of samples (Figure S13c).^39^ For the CPL, however, none of them showed observable signals even after optimizing the gain of the detector and accumulating data over 10 iterations (Figure S13b,d). Compared to other manganese halides showing significant CPL signals, the lack of periodic helical chains as well as the bonding between chiral molecules and manganese cation in (R-/S-PEA)_2_MnBr_4_ may contribute to the low CPL results.^40−43^

To testify to the transition stability between GR–RR and GS–RS states, the thermal cycle measurement was completed inside a N_2_-filled glovebox. As displayed in Figure 2d,e, the PL spectra remained unchanged after 50 cycles. Although there was a 3–10% decrease in PLQY relative to the initial value right after the measurement, a self-recovery process occurred overnight. Since the effect of solvent and oxygen can be neglected inside the N_2_-filled glovebox, we attribute such PLQY variation to ion migration, which has been proven to dominate the degradation and self-recovery processes in other metal halides (Figure 2f).^44−46^ The above results highlight the fully reversible transition processes of GR–RR and GS–RS from a photophysical perspective.

### 3.4 Reusable Time–Temperature Indicator (TTI)

**Figure 3:**
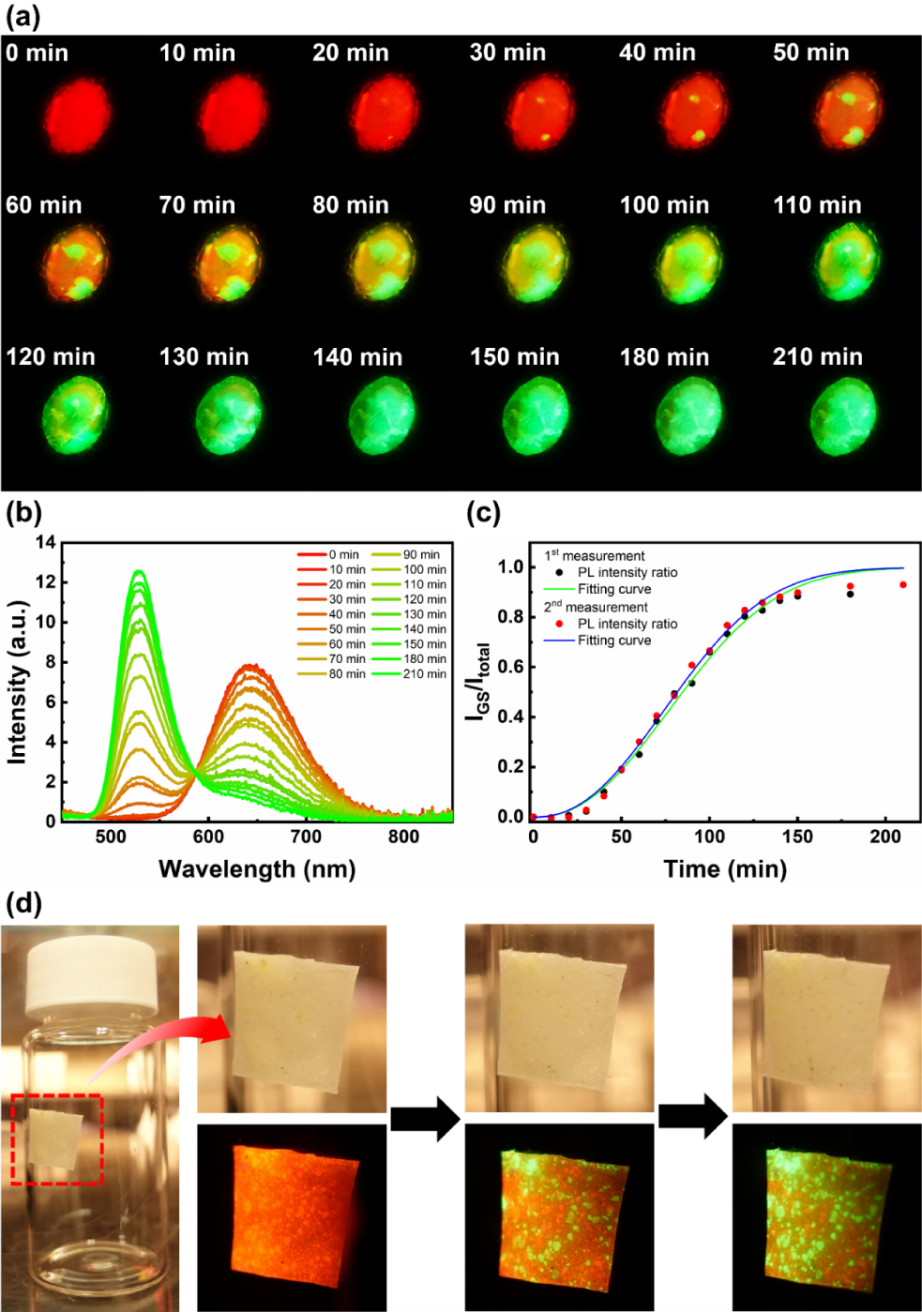
(a) The gradual transition process of the (S-PEA)_2_MnBr_4_ sample from red to green emissive states at 35 °C. (b) The PL spectra of the (S-PEA)_2_MnBr_4_ sample during the transition process. (c) The time-dependent emission intensity ratio along with fitting curves of the first and the second measurements. (d) The transition process of the self-adhesive label under indoor light (top) and UV light (bottom) illumination.

TTIs refer to materials showing continuous variation through redox, diffusion, or phase transition processes at a specific temperature. They can convert the accumulative effects of time and temperature into straightforward visual information and are widely used in monitoring the storage of foods and pharmaceuticals.^47^ Nevertheless, apart from a few materials discovered by Song et al.^48^ and Zhou et al.,^49^ most of the TTIs cannot be reused. In view of both the transition stability and high PLQY of (S-PEA)_2_MnBr_4_, its capability as a reusable TTI was investigated. In Figure 3a, the sequential transition process of the (S-PEA)_2_MnBr_4_ sample turning from red to orange, yellow, and finally green emissive states at 35 °C is demonstrated, and the transition mechanism diagram is presented in Figure S14. It is shown clearly in Figure 3b that the PL intensity at 648 nm steadily decreases, whereas a monotonic increase at 526 nm is observed. Figure S15 depicts the CIE coordination shift from (0.635, 0.353) to (0.313, 0.634), indicating the gradual transition from RS to GS. In order to analyze the transition process quantitatively, the time-dependent ratio of GS emission intensity (*I*_GS_) to the total PL intensity (*I*_total_) was calculated and plotted in Figure 3c. *I*_GS_/*I*_total_ reaches 0.5 at 90 min, and the data points follow a typical sigmoidal curve. Since both crystallization and phase transition were involved in the process, the Avrami equation was selected to be the fitting function:^50^
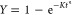
2

where *Y* is the ratio of phase transition volume to the total volume, which is approximated with the PL intensity ratio, *I*_GS_/*I*_total_; *K* is the crystallization rate constant, which depends on the nucleation rate and the growth rate; *t* is the reaction time; and *n* is the dimension of grain growth.^51^ The optimized fitting curve (*R*^2^ = 0.9904) is presented in Figure 3c as the green line, and the fitting parameters *K* and *n* equal to 2.88 × 10^–5^ and 2.27, respectively, representing that the grain growth is two-dimensional. The measured sample was converted back to RS through a melt-quenching process and remeasured subsequently. Unsurprisingly, both data points (red dots) and the fitting curve (blue line, *R*^2^ = 0.9923) show similar trend as prior results, where *K* and *n* equal to 3.08 × 10^–5^ and 2.27, respectively, proving the reusability of (S-PEA)_2_MnBr_4_ TTI. The transition process at 45 °C was measured as well to further illustrate the temperature dependence of (S-PEA)_2_MnBr_4_ TTI (Figure S16). The *K* and *n* were calculated to be 2.84 × 10^–2^ and 2.08, respectively, indicating that the crystallization process is much faster at 45 °C, while the grain growth remains two-dimensional. Similarly, the RR also demonstrated such time-dependent PL transition behavior (Figure S17). Finally, a self-adhesive TTI label was fabricated by mixing PDMS and (S-PEA)_2_MnBr_4_ powders, demonstrating the potential in commercial applications (Figure 3d).

### 3.5 X-ray Scintillator with High Spatial Resolution

**Figure 4:**
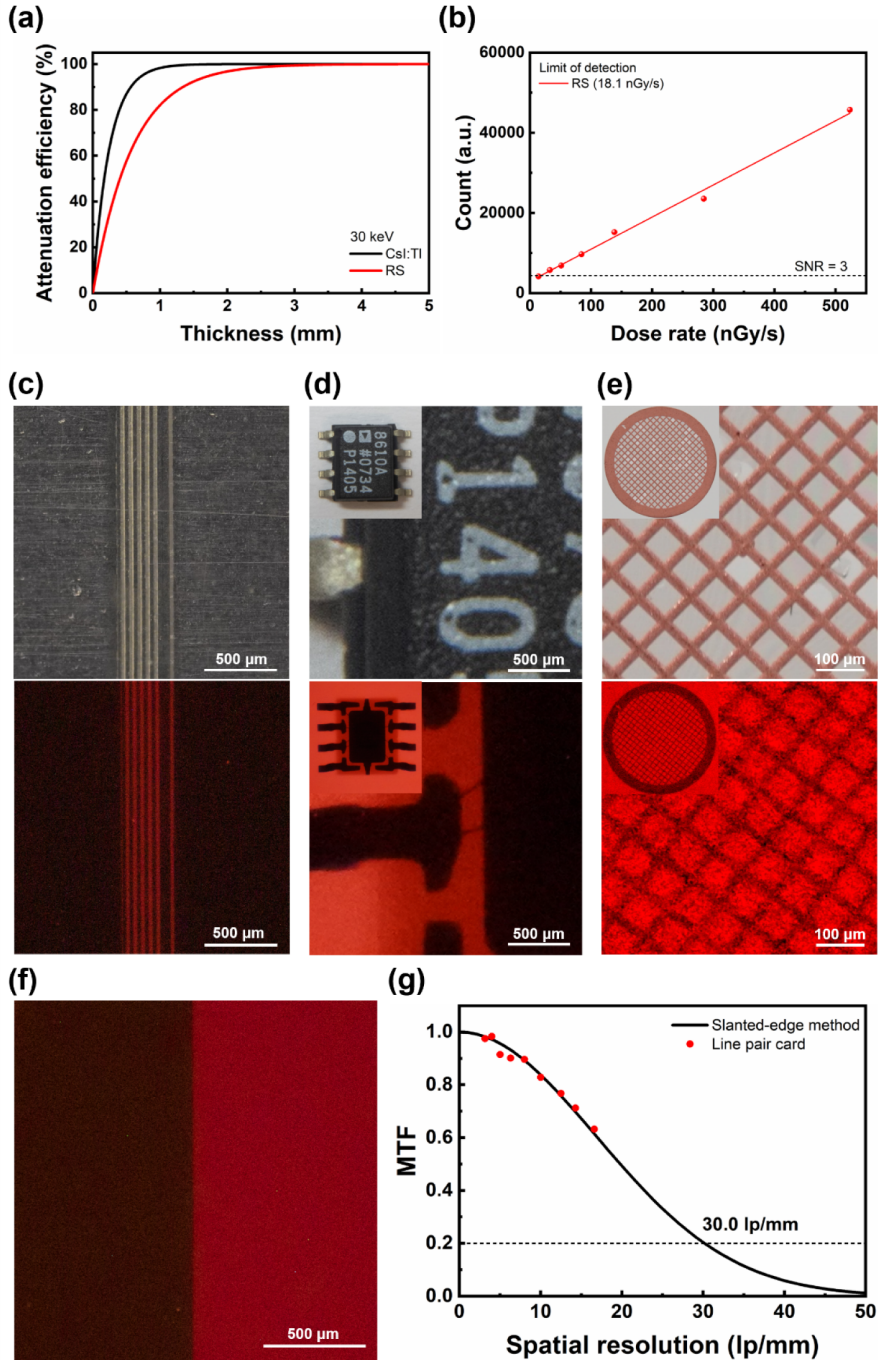
(a) The attenuation efficiency of CsI:Tl and RS to 30 keV X-ray photons. (b) The linear relationship between the X-ray dose rate and the emission intensity of RS. The LoD at SNR = 3 is marked by the dashed line. (c) Images of the 16.6 lp/mm line pair pattern, (d) the IC device and (e) the copper grid under indoor light (top) and X-ray (bottom) illumination. (f) The image used for slanted-edge analysis. (g) The MTF to spatial resolution relationship calculated with the slanted-edge method (black solid line) and line pair card (red dots).

The ultralow haze of 0.0087 (Figure S18) combined with a high PLQY makes RS an ideal candidate for high-resolution X-ray imaging since the suppression of light scattering can greatly improve the spatial resolution ability, which is the most important characteristic of scintillators. First, the radioluminescence spectra were measured and are presented in Figure S19. Subsequently, the X-ray attenuation efficiency of RS and the CsI:Tl reference at 30 keV was calculated and presented in Figure 4a, and the linear relationship between the radioluminescence intensity of RS and the input X-ray dose rate is displayed in Figure 4b. According to the International Union of Pure and Applied Chemistry (IUPAC), the LoD should be defined as the X-ray dose rate at which the signal-to-noise ratio equals 3.^52^ Thus, the LoD of the RS scintillator (thickness: 0.17 mm) was derived as 18.1 nGy/s, far below the requirement for regular medical diagnosis (5500 nGy/s)^53^ and other published manganese halides,^54−57^ while the light yield of RS was estimated to be 3326 photons/MeV, lower than the commercial CsI:Tl scintillator (65 000 photons/MeV). X-ray images of line pair patterns were taken to examine the spatial resolution. As displayed in Figure S20, the images from 3.15 to 14.3 lp/mm can be clearly resolved by the RS scintillator. X-ray images of the 16.6 lp/mm line pair pattern (Figures 4c and S21), the IC device (Figure 4d), and the copper grid (Figure 4e) were further analyzed. All of the details, including bonding wires and copper filaments, were clearly resolved, highlighting the superior resolution of the RS scintillator. We utilized the slanted-edge method (Figures 4f and S22) to obtain the modulation transfer function (MTF) at higher spatial resolutions. As shown in Figures 4g and S23, the resolution limit of the RS scintillator was determined to be 30.0 lp/mm, and the MTF-to-spatial resolution curve below 16.6 lp/mm is in good agreement with data points from the standard line pair card, enhancing the reliability of the experiment. It should be noted that while RS will gradually convert to GS at room temperature, the transition can be suppressed simply by maintaining RS below its glass transition temperature (Figure S24). All in all, the RS scintillator shows not only a low LoD but also remarkable spatial resolution, comparable to other manganese, copper, and lead halides (Table 2).^56,58−67^

**Table 2:** Performance of Manganese, Copper, and Lead Halide X-ray Scintillators

Material	Limit of detection (nGy/s)	Resolution limit (lp/mm)	Reference
(HTTP)_2_MnBr_4_	185	10	(56)
(C_8_H_20_N)_2_MnBr_4_	24.2	4.9	(58)
(HTP)_2_MnBr_4_	130	17.28	(59)
(2-DMAP)_2_MnBr_4_ (thickness: 0.31 mm)	76.35	20–25	(60)
(BTP)_2_MnBr_4_	43.4	14.25	(61)
Cs_3_Cu_2_I_5_–AAO	24.33	10.4	(62)
Cs_3_Cu_2_I_5_:2%In^+^	3582	15	(63)
Cs_5_Cu_3_Cl_6_I_2_@AAO	152	20	(64)
(CuI(pyridine))_4_	55	20	(65)
CsPbBr_3_–CsPb_2_Br_5_	1130	8.19	(66)
CsPbBr_3_	-	15	(67)
(S-PEA)_2_MnBr_4_	18.1	30.0	This work

## 4 Conclusion

In conclusion, highly efficient phosphorescent stimuli-responsive manganese halides, (R-/S-PEA)_2_MnBr_4_, were successfully synthesized through a solvent-free mechanochemical process. The fully reversible kinetically and thermodynamically controlled PL switching between green (λ_max_ = 526 nm) and red (λ_max_ = 648 nm), together with decent PLQYs (green: 91.3%/98.6%, red: 64.9%/79.9% for R-/S-compounds, respectively), was observed for the first time in manganese halides. Utilizing both the high PLQY and fully reversible transition process of (S-PEA)_2_MnBr_4_, reusable TTI and its compatibility with self-adhesive labels were successfully fabricated. Moreover, (S-PEA)_2_MnBr_4_ X-ray scintillators showing a low LoD of 18.1 nGy/s and a high resolution limit of 30.0 lp/mm were developed. This work unveils the versatile properties of (R-/S-PEA)_2_MnBr_4_ and their applicability in the food and medical industries, demonstrating the great potential of manganese halides in various luminescent applications and promoting future research.

## Supporting Information Available

Molecular structures of S-PEABr and R-PEABr, schematic of planetary ball-milling process, appearance of (R-/S-PEA)_2_MnBr_4_, TGA–DSC and LTDSC results, calculated PXRD patterns and temperature-dependent PXRD results, EXAFS of MnBr_2_, additional PLE and absorption spectra, CD and CPL results, CIE coordination, haze measurement results, X-ray images and the intensity distribution of line pair cards, edge spread function and line spread function (Figures S1–S24); detailed crystallographic data, fitting parameters of EXAFS, ligand field splitting energy and Racah parameters (Tables S1–S4) (PDF)
