# Supplementary material for: Highly Efficient Manganese Bromides with Reversible Luminescence Switching through Amorphous–Crystalline Transition
Source: ACS Appl Mater Interfaces. 2024 Oct 3;16(41):55842–51. doi: 10.1021/acsami.4c09396 (PMC11492243; doi:10.1021/acsami.4c09396)
Supplement: Supplementary file 1 — am4c09396_si_001.pdf [file am4c09396_si_001.pdf]

Supporting Information

Highly Efficient Manganese Bromides with Reversible Luminescence Switching through Amorphous–Crystalline Transition

*Guang-Hsun Tan[a], Hao-Cheng Lin[a], Hao-Chi Liang[b], Chih-Wen Pao[c], Po-Yu Chen[d], Wei-Tsung Chuang[c], Chung-An Hsieh[e], Dalia M. Dorrah[b], Ming-Chia Li[f, g], Li-Yin Chen[e], Ho-Hsiu Chou[b] and Hao-Wu Lin[a]**

[a] Department of Materials Science and Engineering, National Tsing Hua University, Hsinchu 30013, Taiwan

[b] Department of Chemical Engineering, National Tsing Hua University, Hsinchu 30013, Taiwan

[c] National Synchrotron Radiation Research Center, Hsinchu 30076, Taiwan

[d] Advanced Packaging Instrumentation and Metrology Laboratory, Industrial Technology Research Institute, Hsinchu 30013, Taiwan

[e] Department of Photonics, College of Electrical and Computer Engineering, National Yang Ming Chiao Tung University, Hsinchu 30010, Taiwan

[f] Department of Biological Science and Technology, College of Biological Science and Technology, National Yang Ming Chiao Tung University, Hsinchu 30010, Taiwan

[g] Center for Intelligent Drug Systems and Smart Bio-devices (IDS2B), Hsinchu 30068, Taiwan

*E-mail: hwlin@mx.nthu.edu.tw (Hao-Wu Lin)

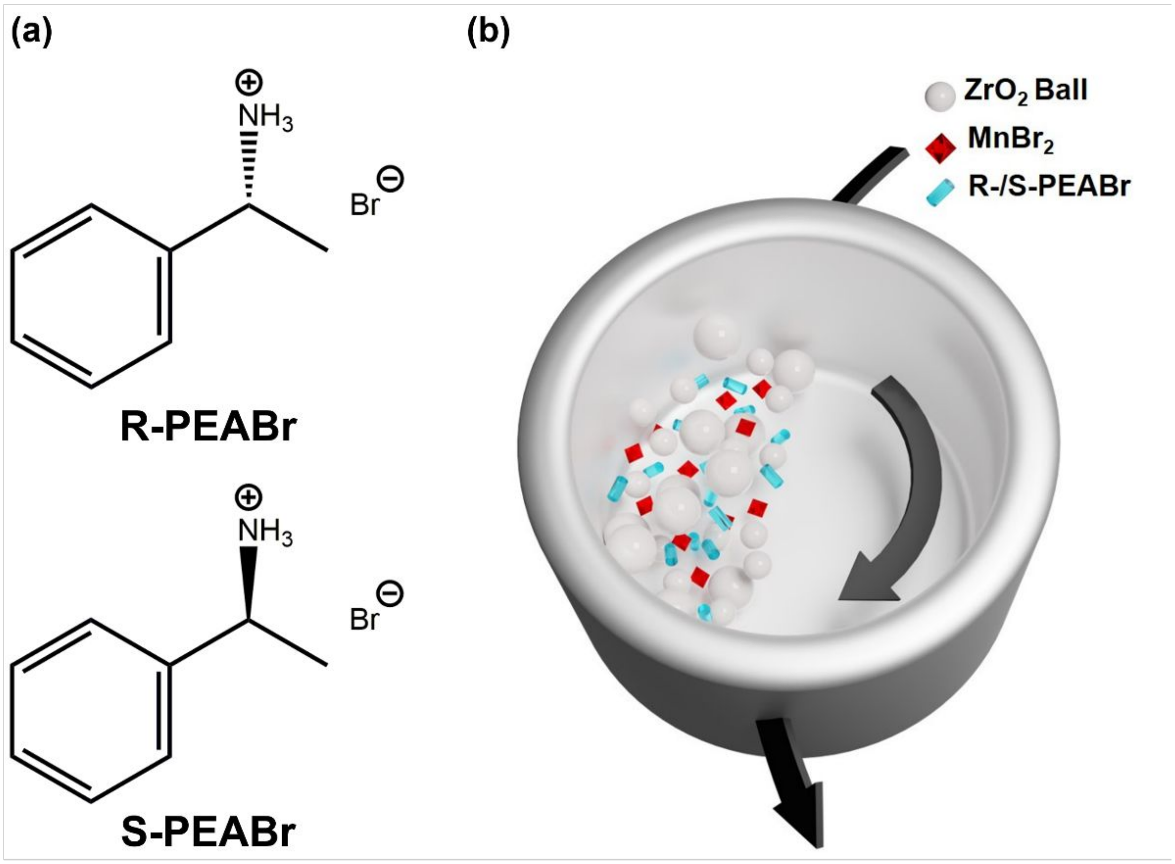

Figure S1. (a) Molecular structures of R-PEABr and S-PEABr. (b) The schematic of planetary ball-milling process.

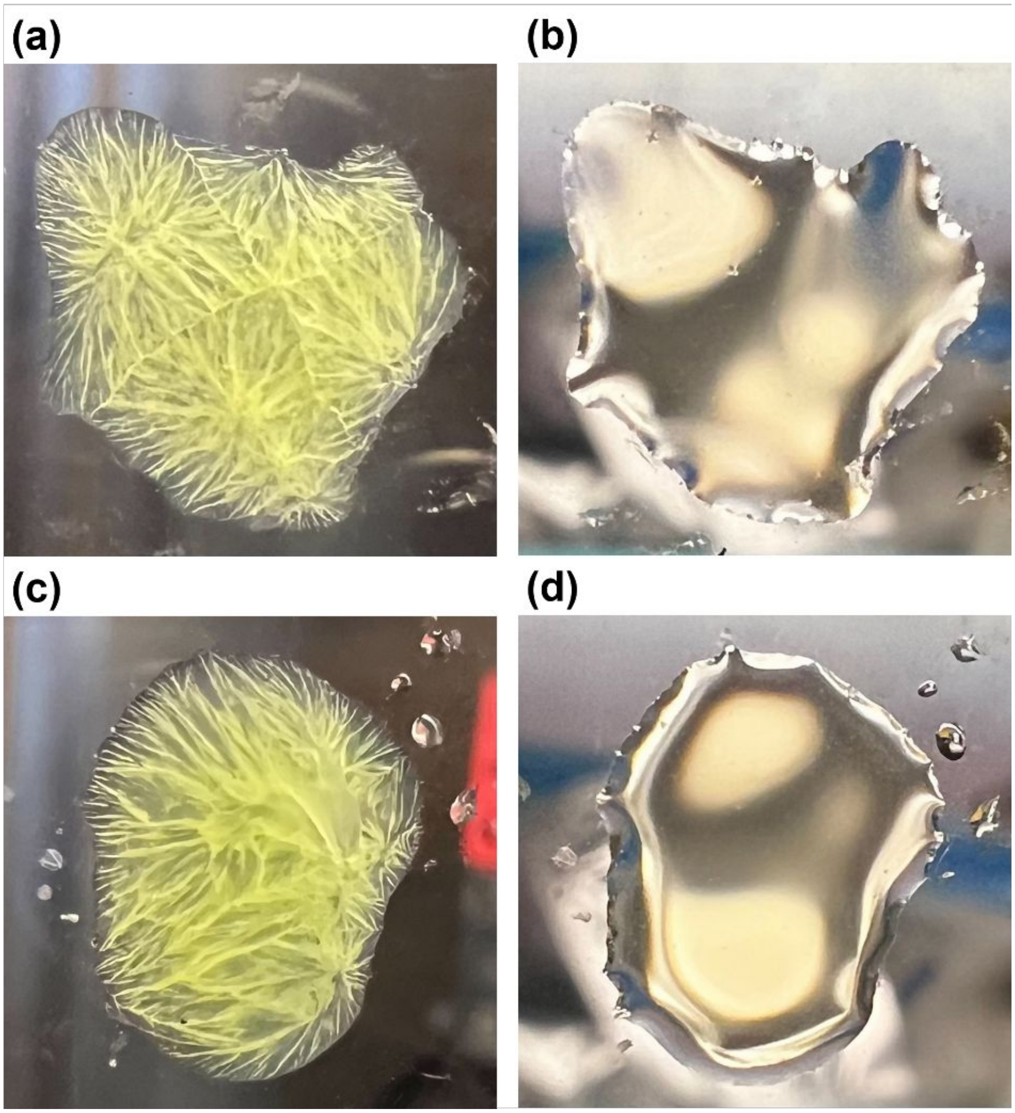

Figure S2. The appearance of (a) GR (b) RR (c) GS (d) RS under indoor light.

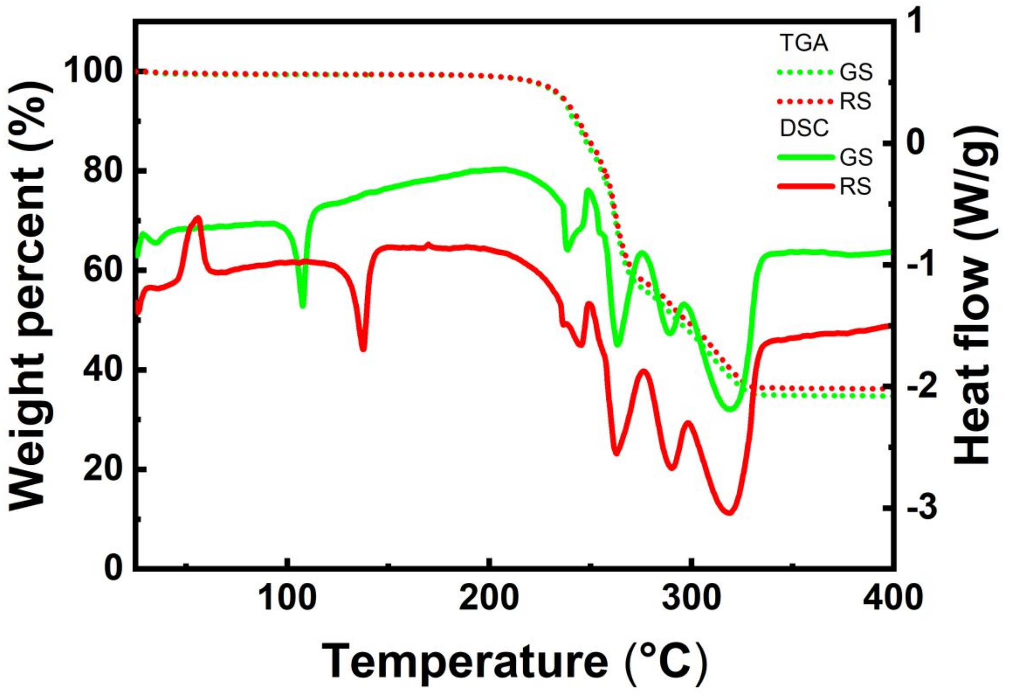

Figure S3. The TGA-DSC measurement of GS and RS.

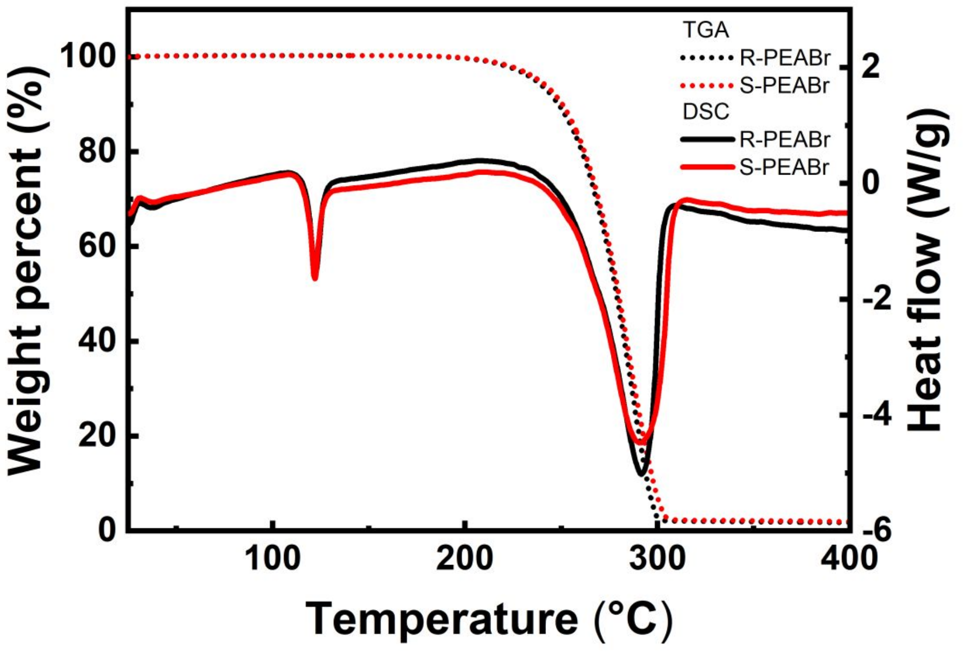

Figure S4. The TGA-DSC measurement of R-PEABr and S-PEABr.

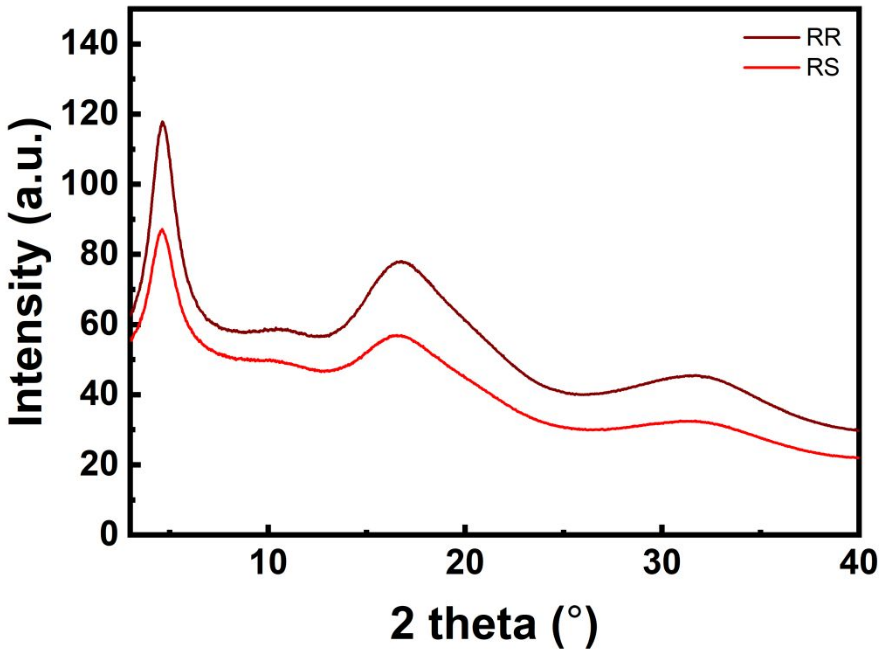

Figure S5. The zoomed-in diffraction patterns of RR and RS.

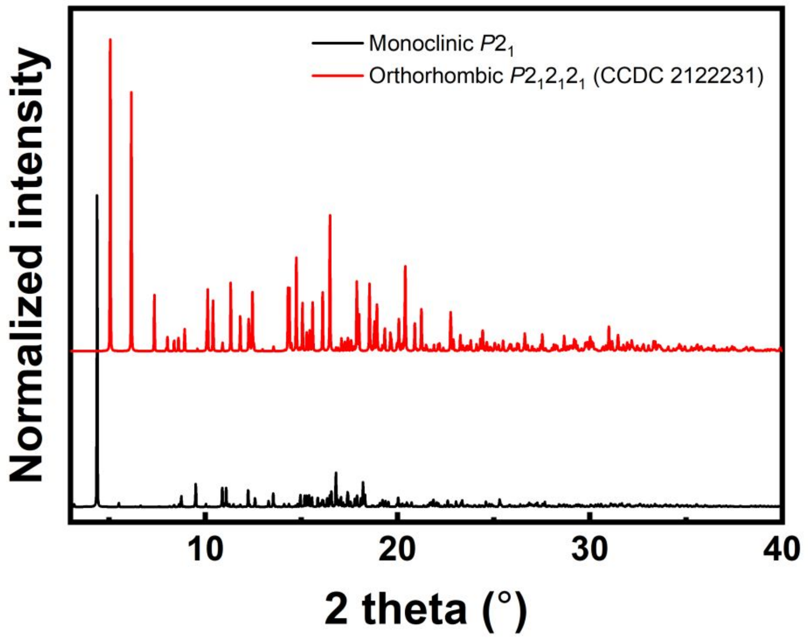

Figure S6. The calculated PXRD patterns (X-ray photon energy: 12 keV) of monoclinic (black) and orthorhombic (red) GR/GS.

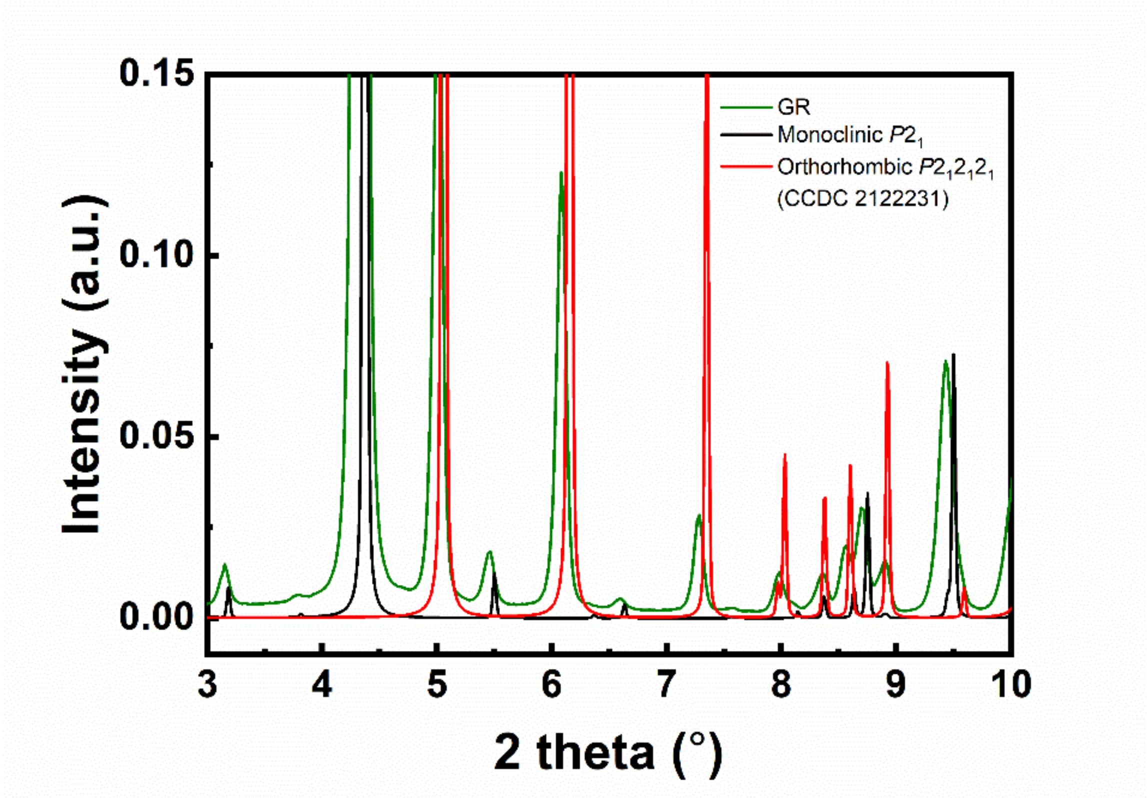

Figure S7. The zoomed-in diffraction peaks of GR powders and the calculated PXRD patterns of monoclinic (black) and orthorhombic (red) structures.

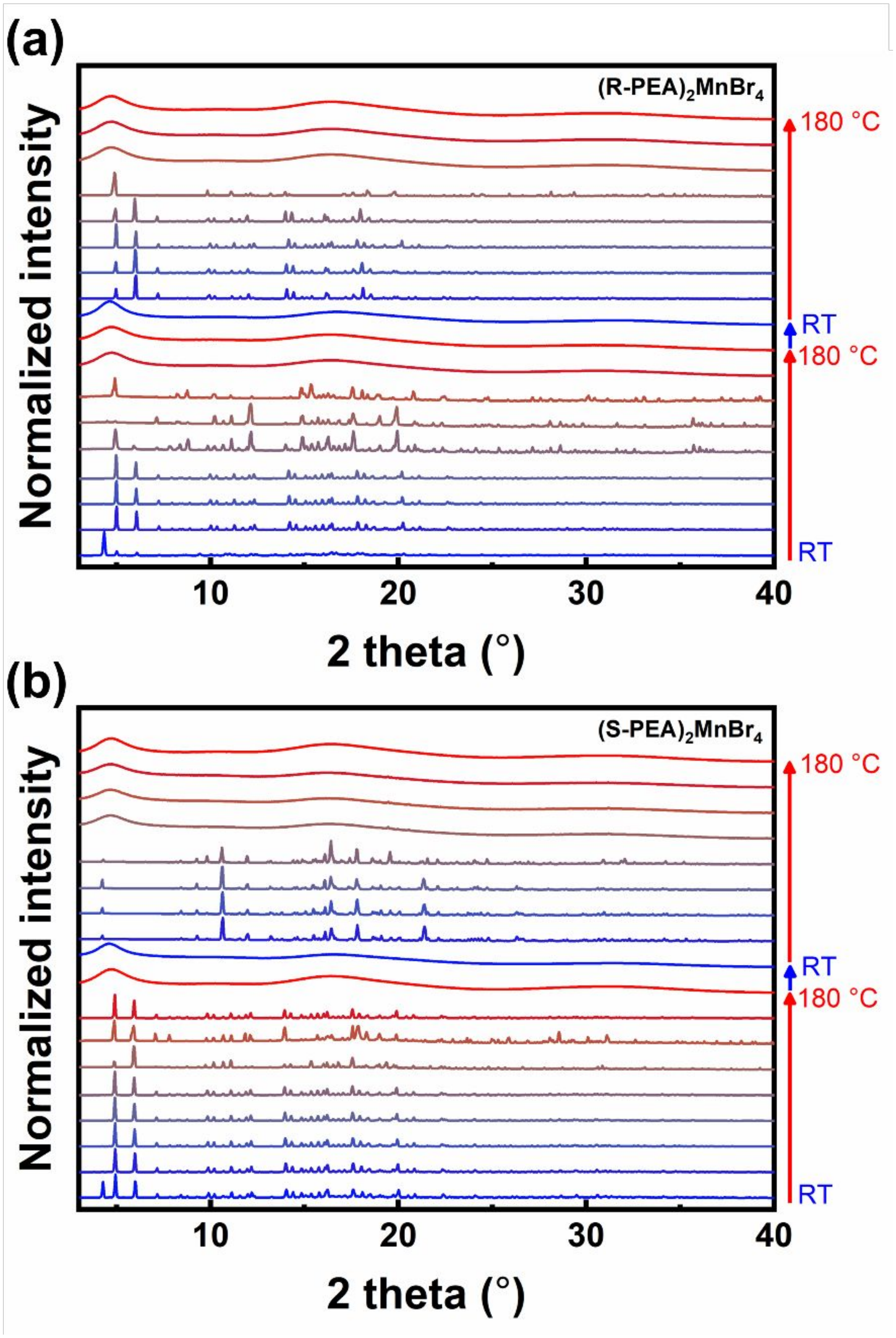

Figure S8. Temperature-dependent PXRD patterns of (a) $(R\text{-}PEA)_2MnBr_4$ (b) $(S\text{-}PEA)_2MnBr_4$.

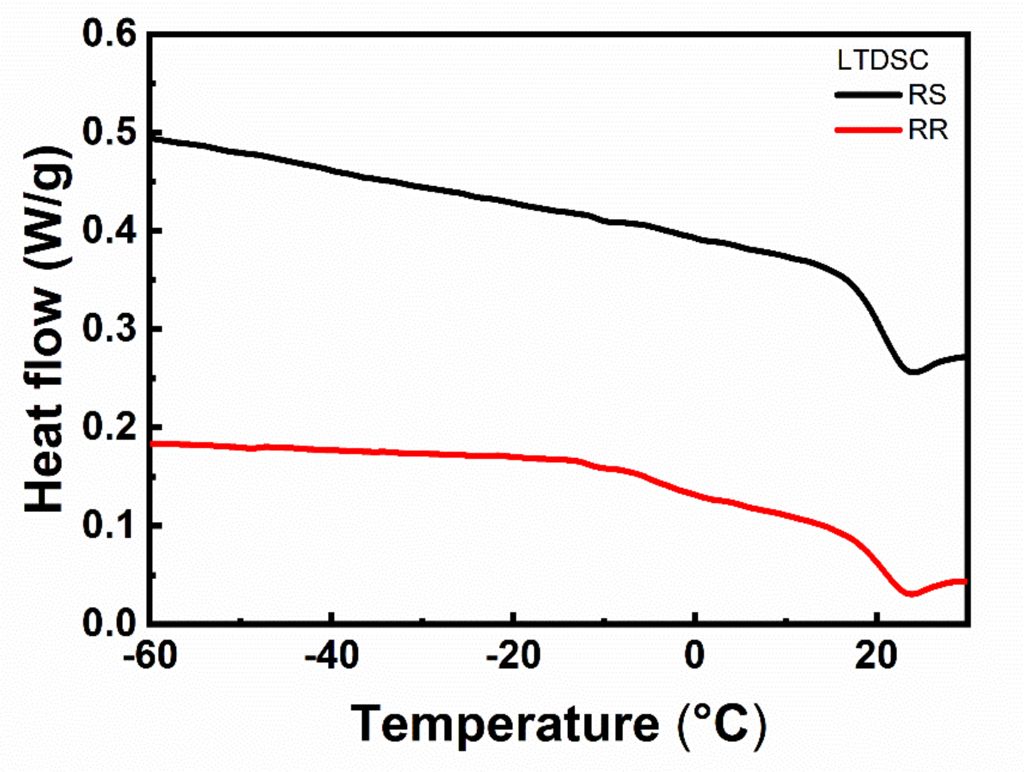

Figure S9. The LTDSC measurement of RS and RR.

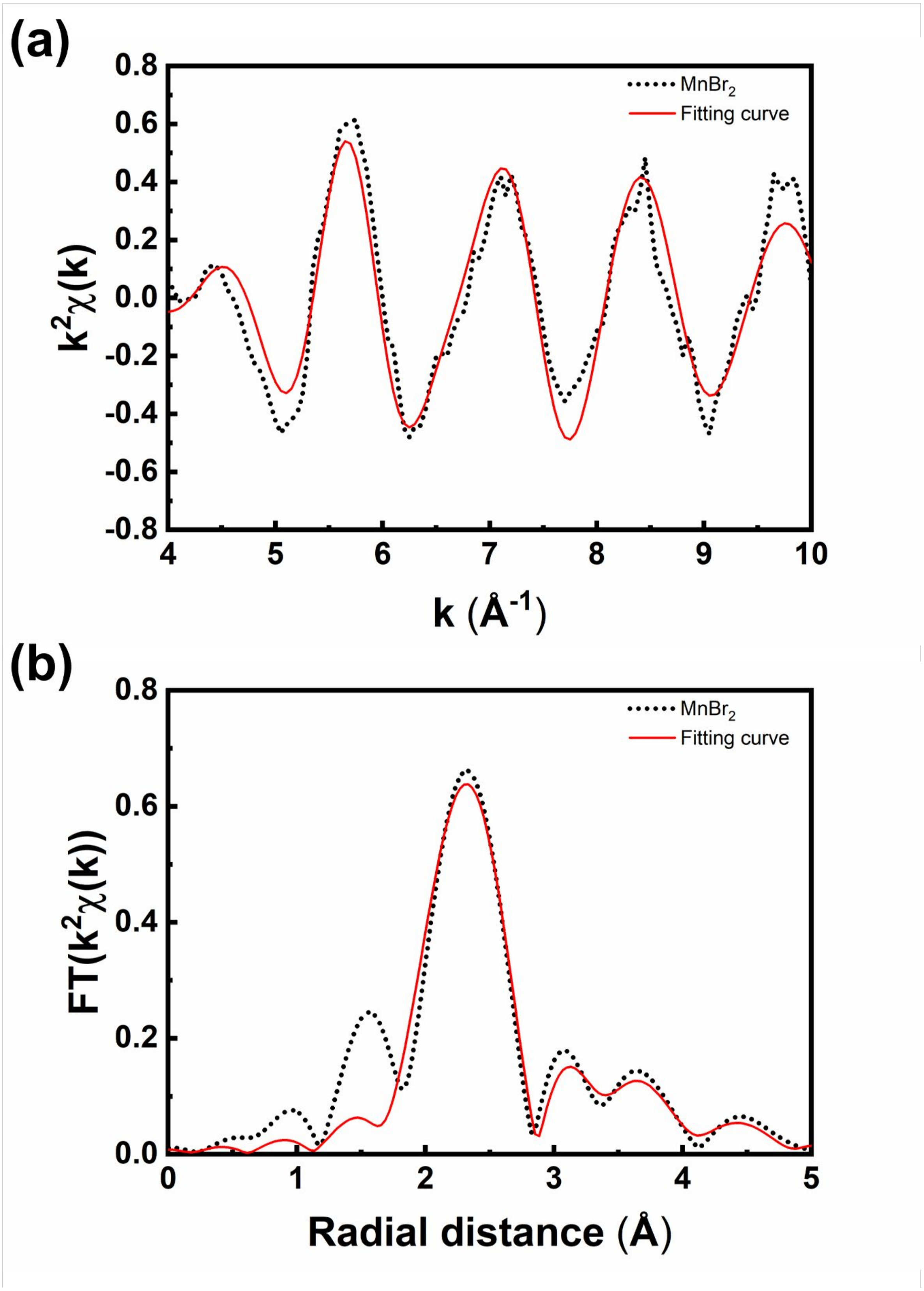

(a)
MnBr2
Fitting curve
k2χ(k)
k (Å-1)
(b)
MnBr2
Fitting curve
FT(k2χ(k))
Radial distance (Å)

Figure S10. (a) The $k^2\chi(k)$ spectra and (b) the Fourier-transformed $k^2\chi(k)$ spectra of $MnBr_2$.

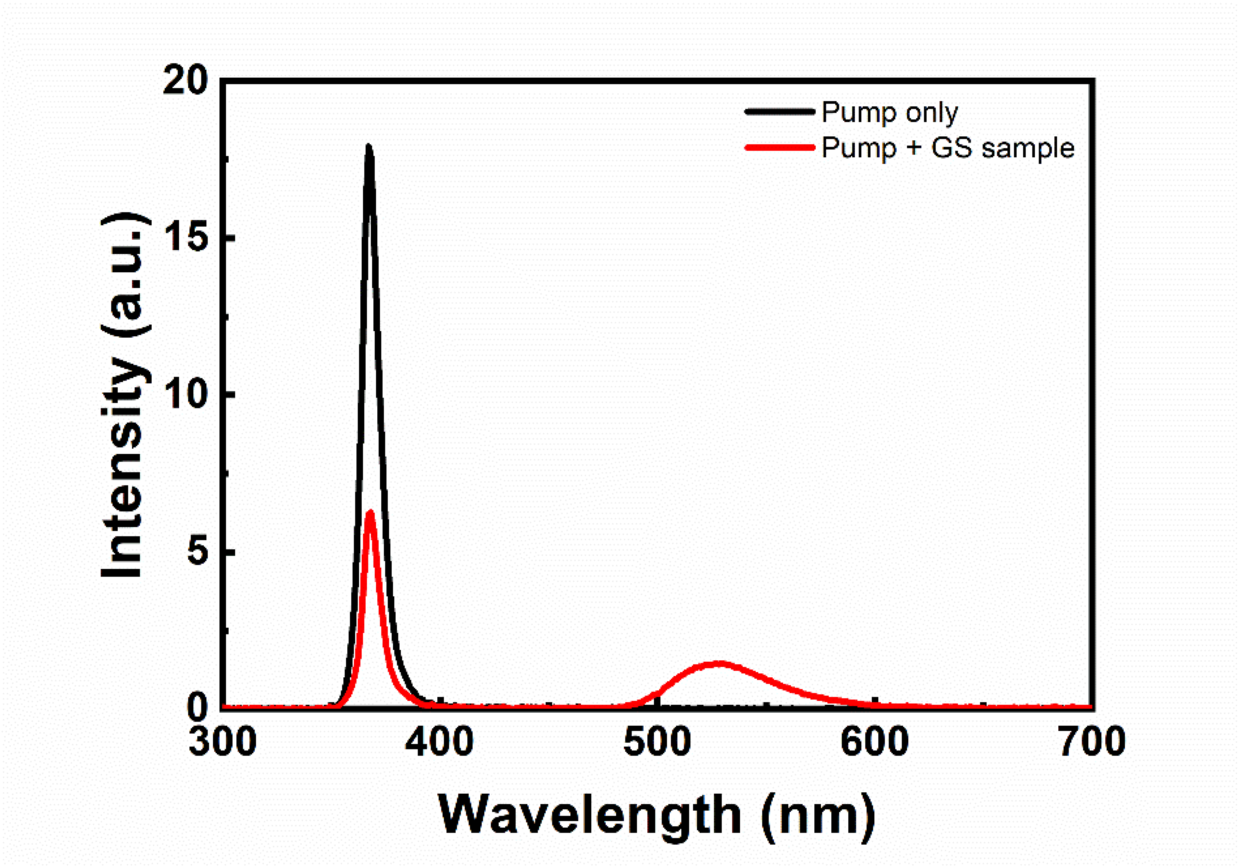

Figure S11. The PLQY spectra of GS.

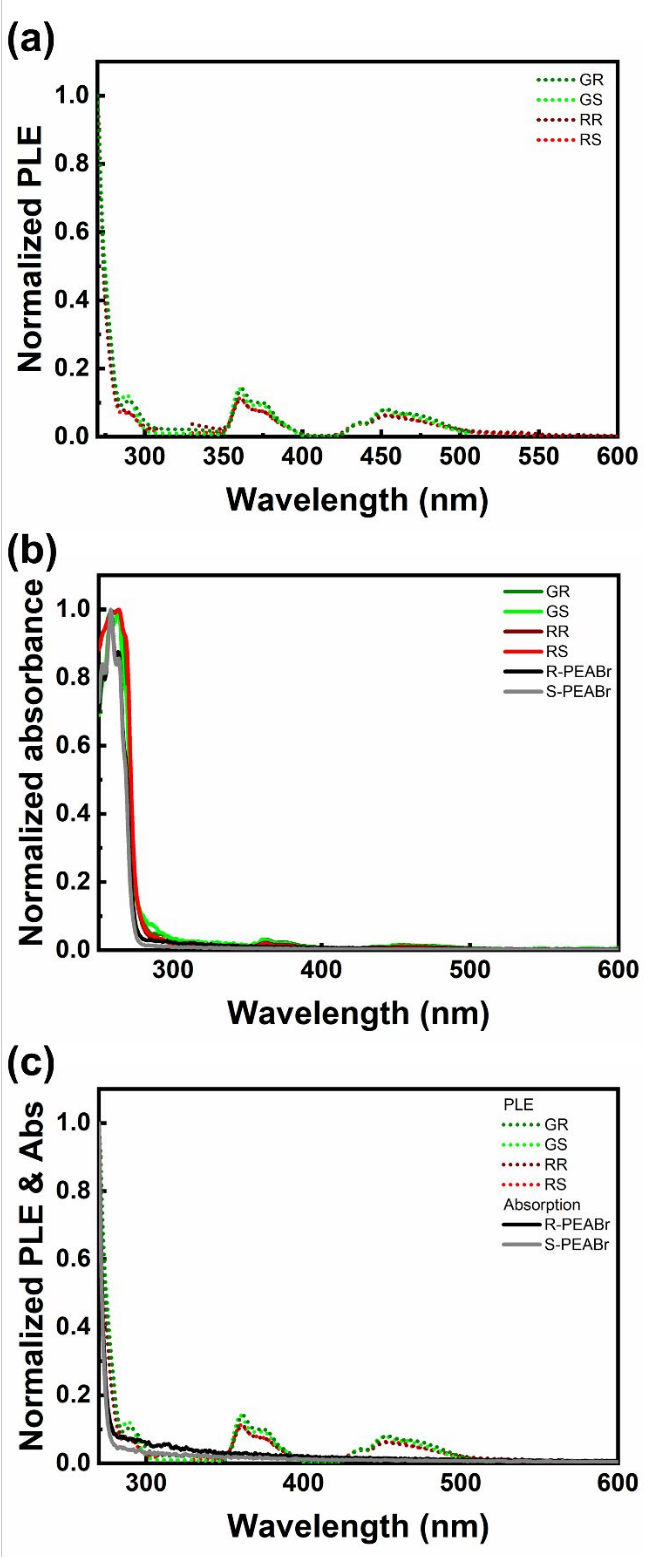

Figure S12. (a) The PLE spectra of GR, GS, RR and RS. (b) The absorption spectra of manganese bromides (GR, GS, RR, RS) and precursors (R-PEABr and S-PEABr). (c) The comparison of PLE spectra with absorption spectra under the same wavelength range.

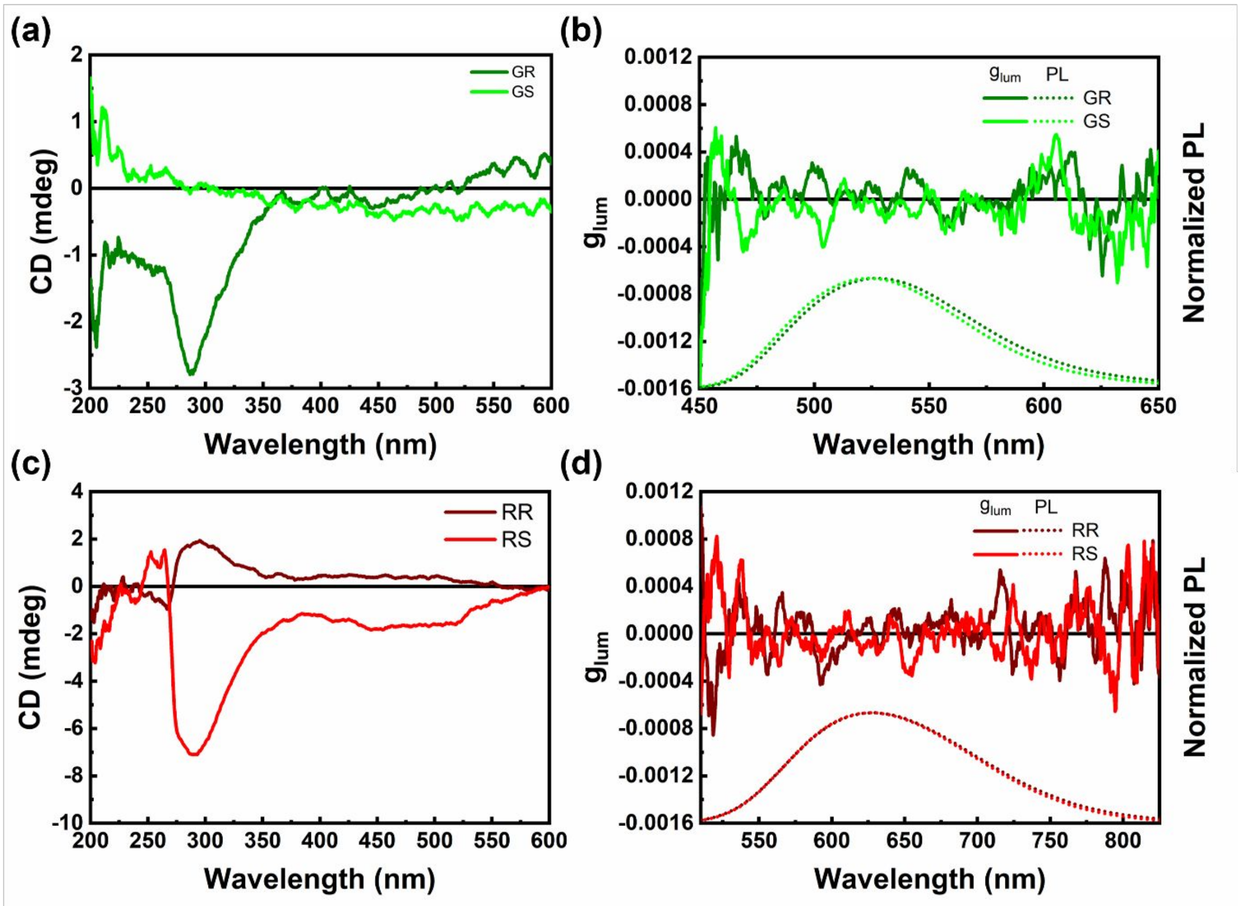

Figure S13. The CD spectra of (a) GR and GS (c) RR and RS together with the CPL spectrum and g_{lum} of (b) GR and GS (d) RR and RS.

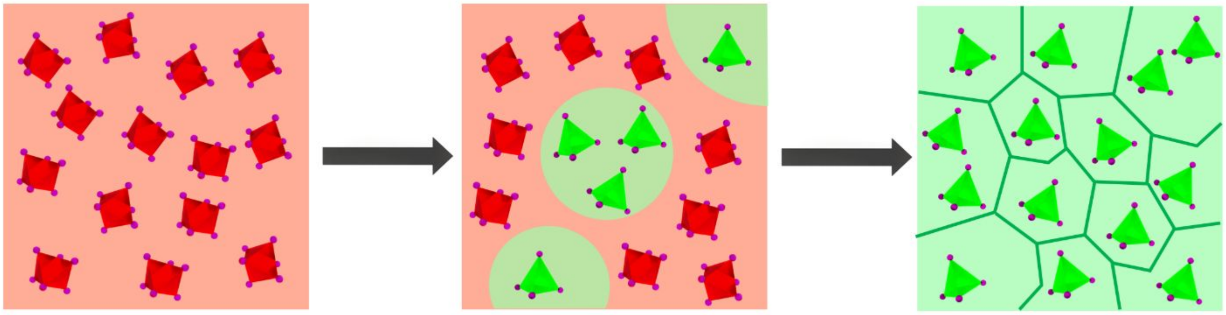

Figure S14. The transition mechanism of the time-temperature indicator involves several stages. Initially, the six-coordinated Mn^{2+} ions are distributed uniformly inside the amorphous (R-/S-PEA)$_2$MnBr$_4$ right after the melt-quenching process. However, part of the material will gradually transform into a crystalline state which consists of four-coordinated Mn^{2+} ions at elevated temperature. Finally, most of the (R-/S-PEA)$_2$MnBr$_4$ will transform into a crystalline state with four-coordinated Mn^{2+} ions after a longer period of time.

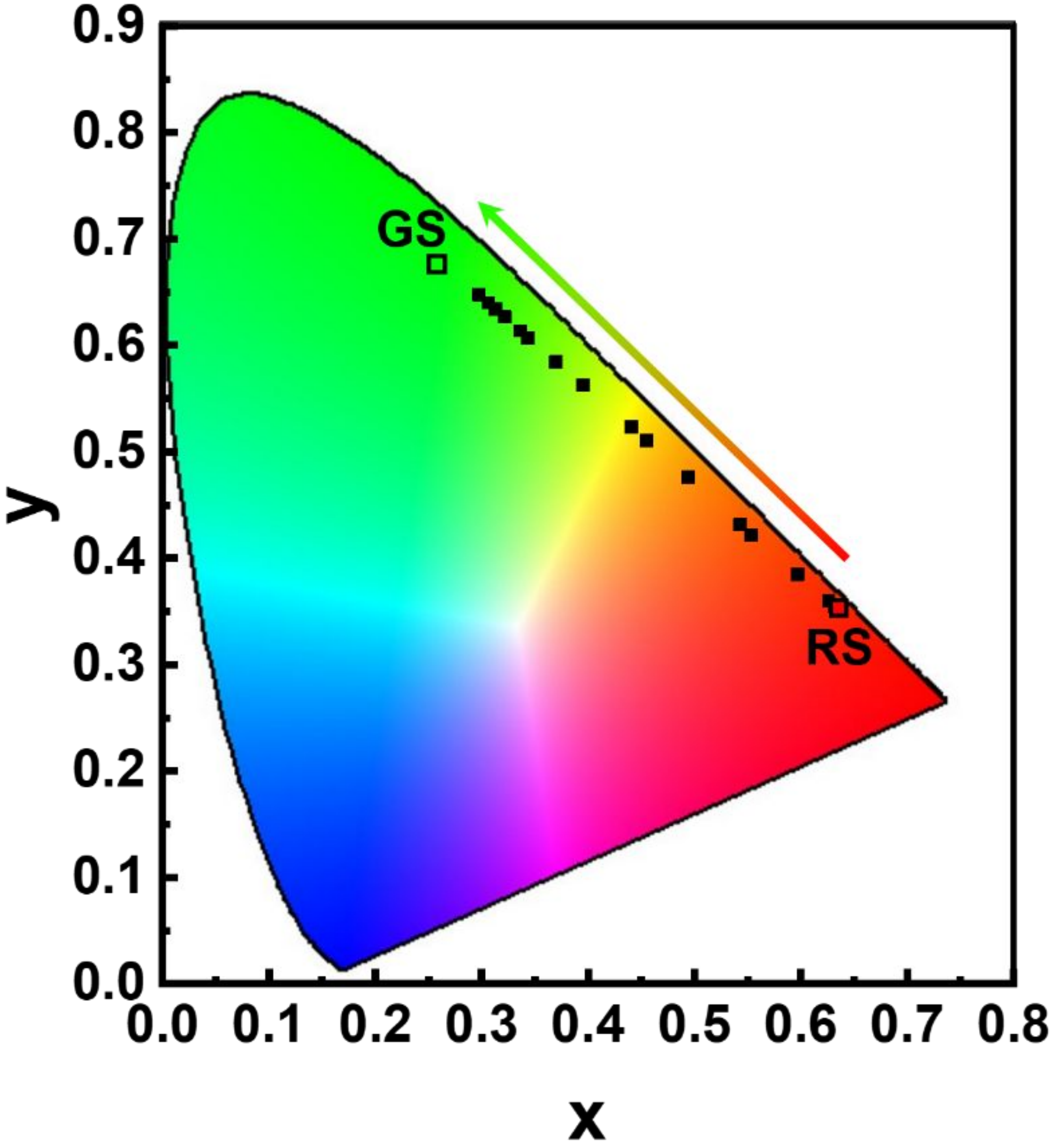

Figure S15. The CIE coordination shift during RS-to-GS transition.

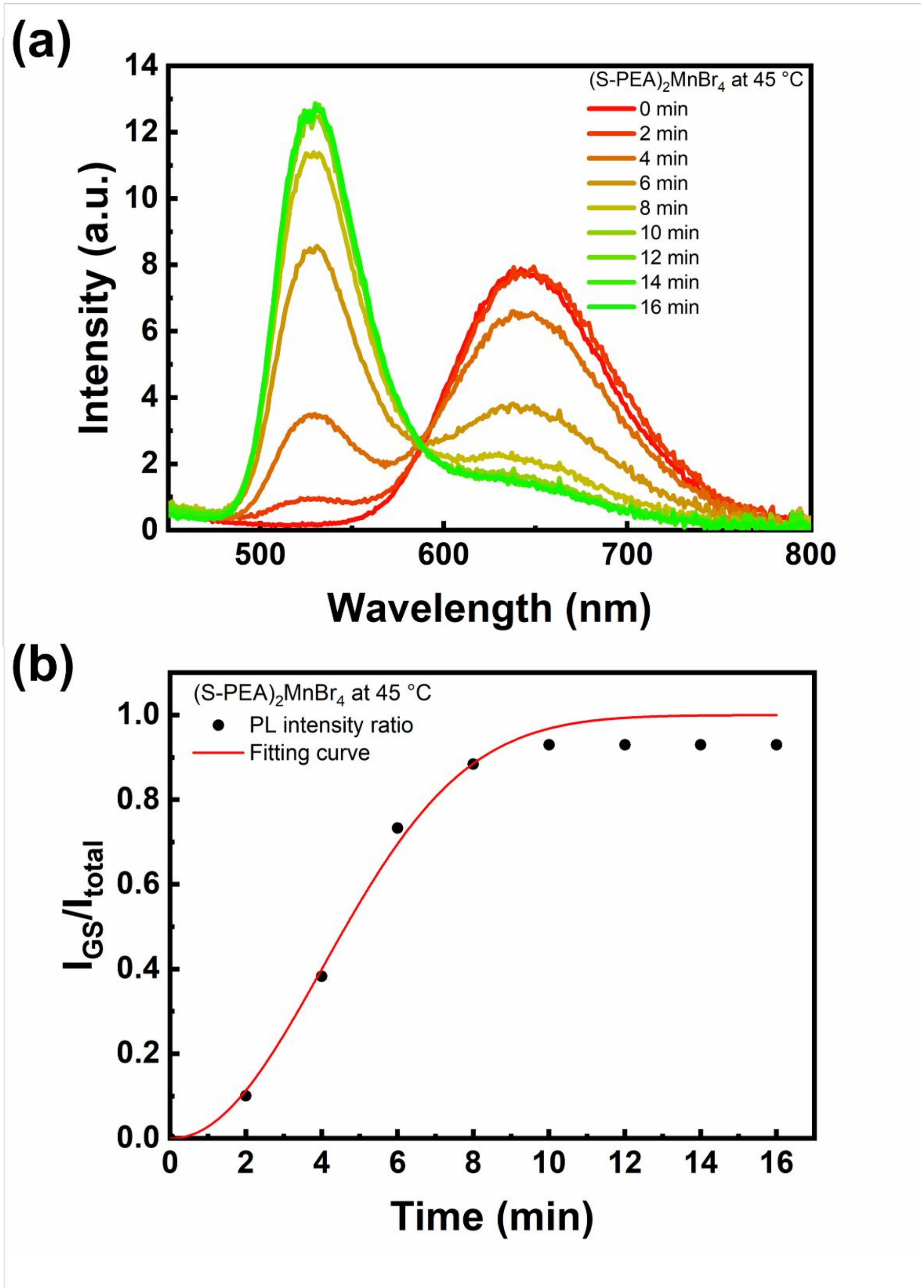

Figure S16. (a) The time-dependent PL spectra of $(S\text{-}PEA)_2MnBr_4$ at 45 °C. (b) The time-dependent emission intensity ratio at 45 °C.

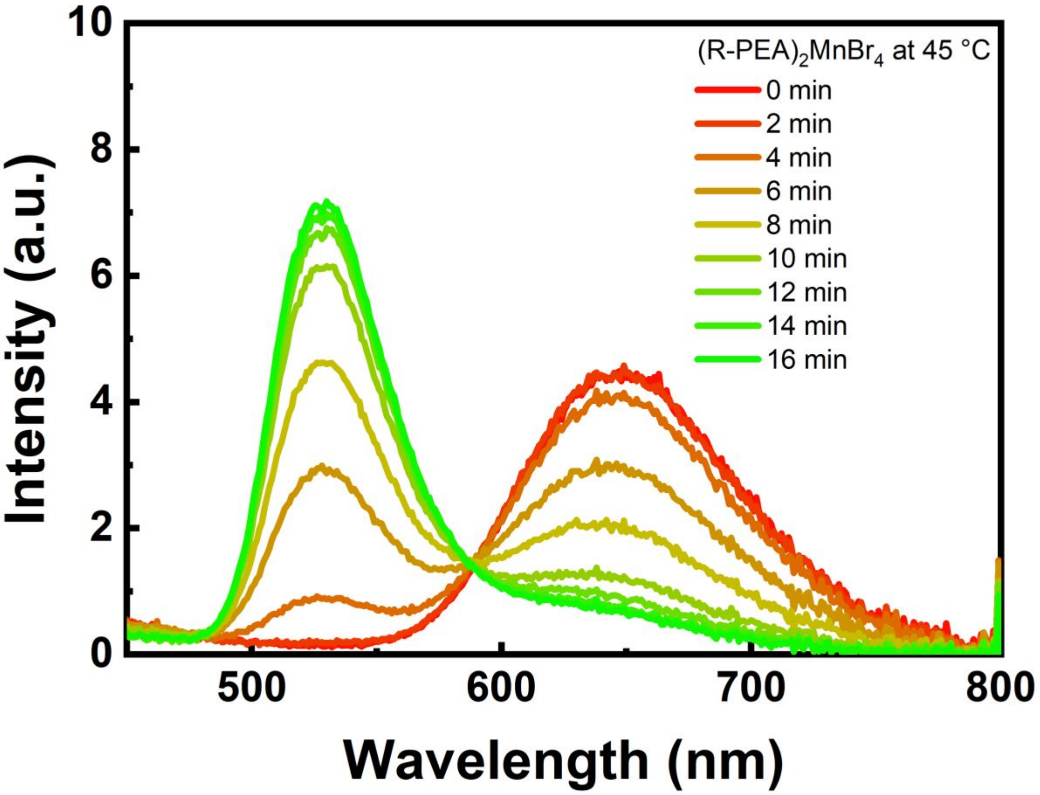

Figure S17. The time-dependent PL spectra of $(R\text{-}PEA)_2MnBr_4$ at 45 °C.

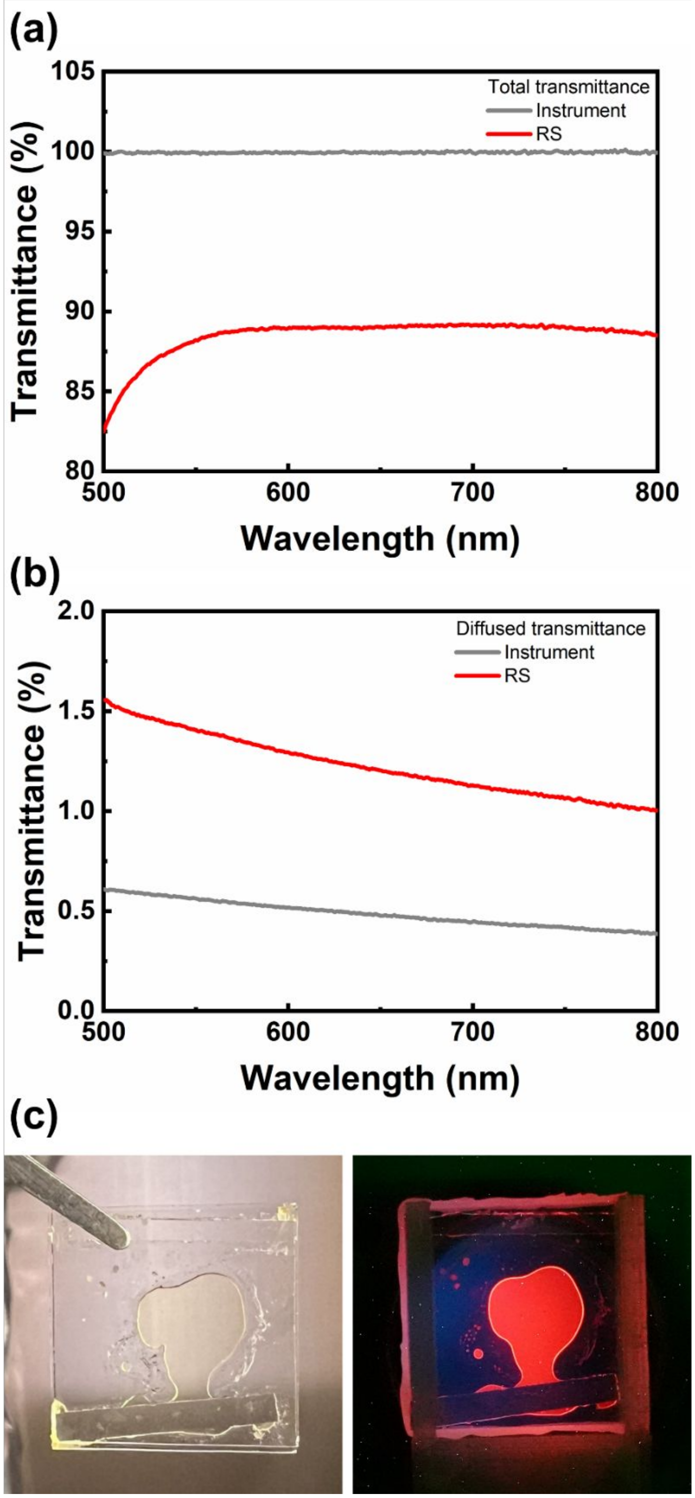

(a)
Total transmittance
Instrument
RS
Transmittance (%)
Wavelength (nm)
(b)
Diffused transmittance
Instrument
RS
Transmittance (%)
Wavelength (nm)
(c)

Figure S18. (a) The total transmittance and (b) the diffused transmittance of the RS sample with 170 μm thickness. (c) The appearance of RS sample under indoor light (left) and X-ray (right) illumination.

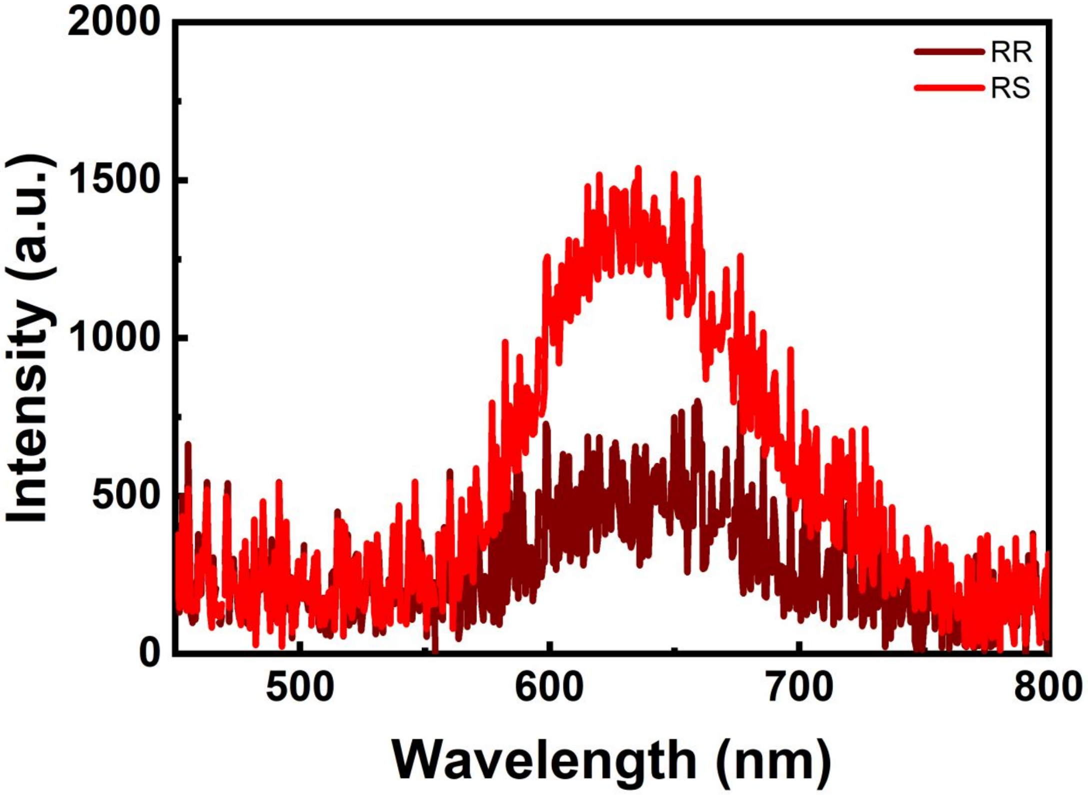

Figure S19. The RL spectra of RR and RS.

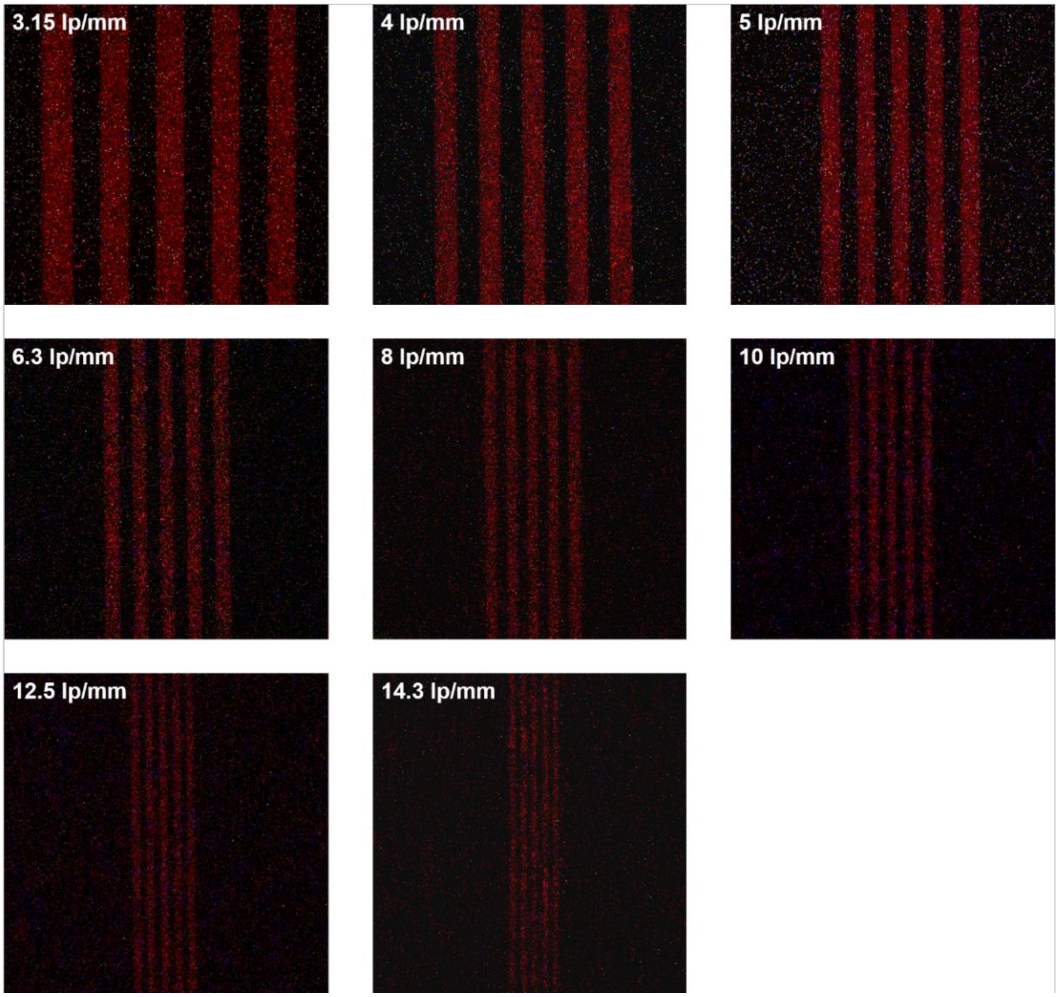

Figure S20. The X-ray images of line pairs ranging from 3.15 lp/mm to 14.3 lp/mm.

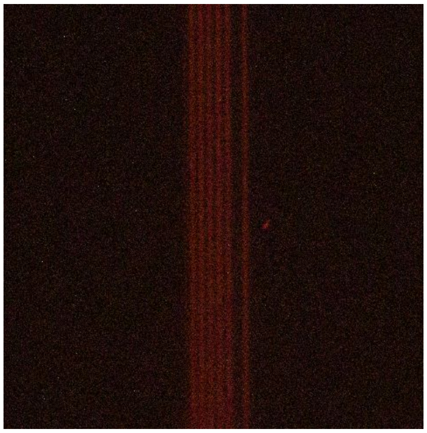

Figure S21. The 16.6 lp/mm line pairs X-ray image of the RR scintillator.

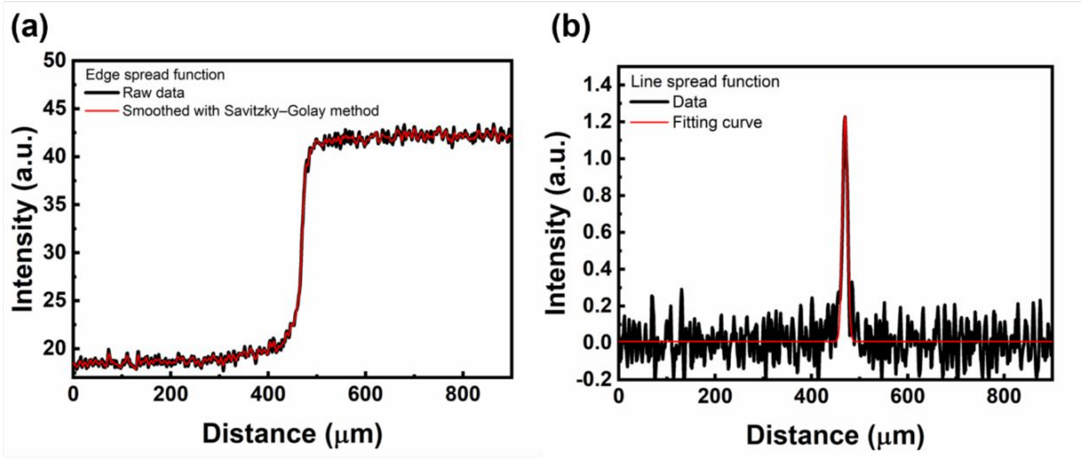

Figure S22. (a) The edge spread function and (b) the line spread function of RS scintillator by slanted-edge method.

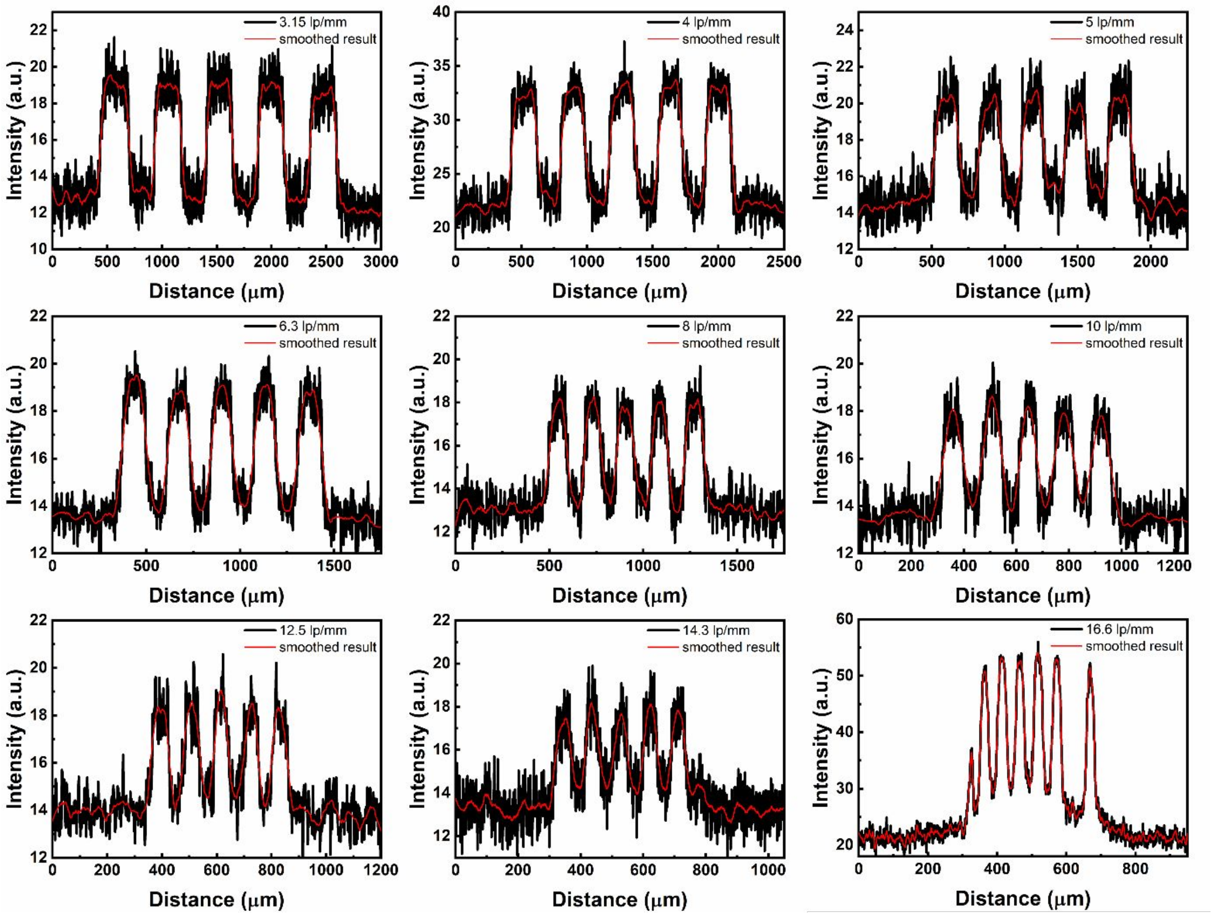

Figure S23. The intensity distribution of the X-ray images of line pair cards ranging from 3.15 lp/mm to 14.3 lp/mm.

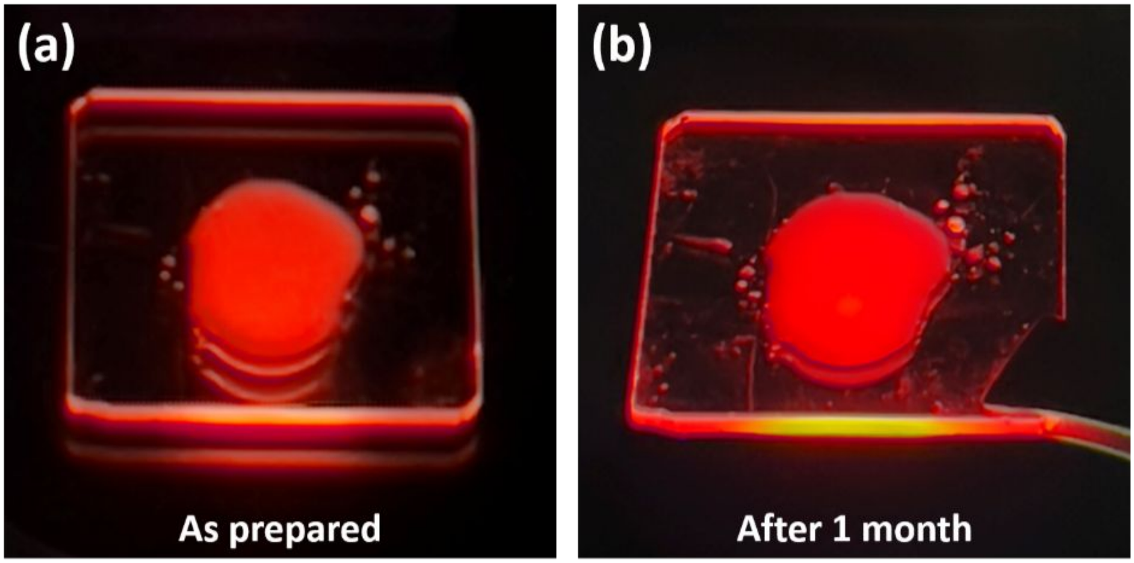

Figure S24. The appearance of (a) as-prepared RS and (b) RS preserved at 18˚C for 1 month. There's no observable green emission, indicating that the amorphous-to crystalline transition is suppressed.

Table S1. The crystallographic data of monoclinic $(R\text{-}PEA)_2MnBr_4$.

Material	$(R\text{-}PEA)_2MnBr_4$
Measuring temperature (K)	100
Empirical formula	$C_{16}H_{24}Br_4MnN_2$
Formula weight	618.95
Crystal system	monoclinic
Space group	$P2_1$
a (Å)	15.9510(5)
b (Å)	7.6532(2)
c (Å)	19.1058(6)
α (°)	90
β (°)	103.247(3)
γ (°)	90
Volume (Å^3)	2270.30(12)
Z	2
ρ_{calc} (g cm^{-3})	1.811
μ (mm^{-1})	12.967
F(000)	1196.0
Goodness-of-fit on F2	1.072
Final R indexes [I>=2σ (I)]	R1 = 0.0571<br>wR2 = 0.1440
Final R indexes [all data]	R1 = 0.0652<br>wR2 = 0.1487

Table S2. The detailed bond lengths and bond angles of monoclinic $P2_1$ and orthorhombic $P2_12_12_1$ $(R\text{-}PEA)_2MnBr_4$.

$(R\text{-}PEA)_2MnBr_4$		
Space group	$P2_1$	$P2_12_12_1$
Bond length (Å)		
Mn1-Br1	2.494(3)	2.4946(12)
Mn1-Br2	2.482(3)	2.5232(13)
Mn1-Br3	2.529(3)	2.4755(11)
Mn1-Br4	2.486(4)	2.5492(12)
Bond angle (°)		
Br1-Mn1-Br2	107.64(12)	101.30(4)
Br1-Mn1-Br3	110.36(12)	113.99(5)
Br1-Mn1-Br4	108.01(12)	109.63(5)
Br2-Mn1-Br3	111.70(12)	119.10(5)
Br2-Mn1-Br4	109.86(13)	104.26(5)
Br3-Mn1-Br4	109.16(12)	107.90(4)

Table S3. The fitting parameters used in the EXAFS analysis of $MnBr_2$ standard.

Material	Scattering path[a)]	S_0^2	N	r (Å)	ΔE_0 (eV)	σ^2	R-factor
	Mn_0-Br_1-Mn_0			2.65		0.011	
$MnBr_2$	Mn_0-Mn_1-Mn_0	0.59	6 (fixed)	3.89	-4.64	0.023	0.016
	Mn_0-Br_2-Mn_0			4.76		0.022	

a) The subscripts 0, 1 and 2 indicate the scattering center, the first scattering shell and the second scattering shell respectively.

Table S4. The ligand field splitting energy and the Racah parameter of GR, GS, RR and RS.

Material	Δ/B	Δ (cm^{-1})	B (cm^{-1})
GR/GS	4.7	3314.6	705.2
RR/RS	9.4	6679.6	710.6
